# Continuous discovery of novel 2D materials via dual active learning-driven generative models

**DOI:** 10.1093/nsr/nwag101

**Published:** 2026-02-12

**Authors:** Xinyu Chen, Zhilong Song, Shuaihua Lu, Qian Chen, Yuanqiu Mo, Qionghua Zhou, Jinlan Wang

**Affiliations:** Key Laboratory of Quantum Materials and Devices of Ministry of Education, School of Physics, Southeast University, Nanjing 211189, China; Suzhou Laboratory, Suzhou 215004, China; Key Laboratory of Quantum Materials and Devices of Ministry of Education, School of Physics, Southeast University, Nanjing 211189, China; Key Laboratory of Quantum Materials and Devices of Ministry of Education, School of Physics, Southeast University, Nanjing 211189, China; Jiangsu Provincial Key Laboratory of Networked Collective Intelligence, School of Mathematics, Southeast University, Nanjing 211189, China; Key Laboratory of Quantum Materials and Devices of Ministry of Education, School of Physics, Southeast University, Nanjing 211189, China; Suzhou Laboratory, Suzhou 215004, China; Key Laboratory of Quantum Materials and Devices of Ministry of Education, School of Physics, Southeast University, Nanjing 211189, China; Suzhou Laboratory, Suzhou 215004, China

**Keywords:** active learning, generative model, materials discovery, two-dimensional materials, density functional theory calculation

## Abstract

Generative artificial intelligence is transforming materials discovery by creating unexplored candidates. However, such models are trapped in historical data bias, particularly for data-scarce systems like two-dimensional (2D) materials, leading to repetitive outputs rather than genuine discoveries. Here, we introduce DuALGen, a dual active learning framework that mitigates these limitations. DuALGen couples two complementary loops to enrich data diversity and correct data bias: a generative loop that uses dynamic, multi-criteria sampling to drive exploration of the design space, and a predictive loop that samples outliers to counter distribution shift, enabling reliable evaluation of novel, previously unknown candidates. Applied to 2D materials, DuALGen uncovers >10 000 stable, distinct compounds, including thousands of high-performance candidates for electronic applications. This self-updating workflow connects generative models to uncharted chemical spaces, and offers a practical route to continuous discovery of new materials.

## Teaser

This study introduces DuALGen, which couples generative and predictive active learning loops to explore unknown chemical spaces, discovering >10,000 novel, stable 2D materials with high-performance candidates for electronic applications.

## INTRODUCTION

Generative artificial intelligence (AI) is driving a paradigm shift across various fields, from chatbots to image creators [1]. This shift is also profoundly reshaping materials design, unlocking new opportunities for exploring materials previously unimaginable [2–6]. However, the novelty of AI-generated materials is facing growing skepticism, as many candidates turn out to already exist or even appear in the models’ own training dataset [7]. This limitation stems from the inherent scarcity of materials data, which covers only a small fraction of the vast chemical space [8], and thus generative models trained on such sparse data struggle to effectively explore the full material landscape [9,10]. Moreover, materials data is often biased. In emerging systems like two-dimensional (2D) materials, only a limited number of stable compounds, largely derived from elemental substitutions, have been computationally identified. Such scarcity and bias limit the scope of available training data, confining generative models to a narrow design space, hindering their extrapolative ability to discover novel materials with transformative potentials [11].

An intuitive solution to overcome data limitations is to retrain the generator using generated data, creating a ‘perpetual generation machine’ that continuously explores novel materials. While appealing given the availability of AI-generated materials in established databases like C2DB [12,13], this retraining approach often suffers from mode collapse [14,15]. In materials discovery, this manifests in two ways. First, models may generate candidates that closely resemble known compounds, reinforcing data bias and producing repetitive outputs. Second, they can generate candidates that fall outside reasonable chemical boundaries, resulting in unstable structures. To avoid these issues and make this retraining strategy actually viable, it is crucial to carefully design sampling strategies that ensure both efficient and reliable exploration.

Existing sampling strategies often focus on a single dominant objective, with simple and fixed criteria that fail to evolve as new chemical space is explored. For example, generative models have been successfully applied to design materials with specific properties [5], compositions [2] and symmetries [16]. However, these models prioritize meeting predefined conditions, often overlooking the novelty and diversity of the generated materials. In these works, AI-based energy predictors [17] are commonly used to evaluate the stability of generated materials, which are typically trained on existing materials. As a result, these predictors inherently carry biases and struggle to evaluate truly novel materials that lie outside their training distribution, leading to inaccurate stability assessments. To address these limitations, a comprehensive, self-updating sampling strategy that is capable of driving generative models to explore uncharted chemical spaces continuously [18–20] is urgently needed.

In this work, we develop DuALGen, a generative framework augmented by dual active learning for continuous model refinement and efficient materials discovery. DuALGen features two synergistic AL loops: a generative loop that employs multi-criteria sampling to enrich training data and expand the design space, and a predictive loop that selects out-of-distribution samples to update all predictors in the generative workflow. To support both loops, DuALGen introduces evolving evaluation that updates thermodynamic phase diagrams and latent space distributions as new chemical spaces are explored, enabling adaptive, comprehensive assessments. As a successful demonstration, DuALGen sustains exploration of novel, stable compounds while delivering accurate property predictors, identifying >1000 candidates with exceptional functional properties. This combination of continuous material generation and accurate property prediction facilitates efficient and scalable exploration of unknown design spaces and opens avenues for materials discovery beyond established knowledge.

## RESULTS

### Workflow of DuALGen

**Figure 1.**
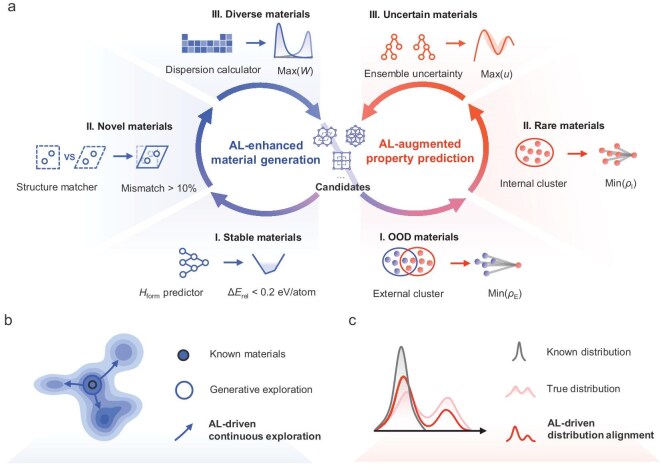
Dual active learning generative framework (DuALGen) for continuous discovery of 2D materials. (a) DuALGen integrates two AL loops: the first loop uses known stable 2D materials to train a generative model, evaluating generated candidates for stability, novelty, and diversity via energy predictors and structural matchers. High-scoring candidates are validated via density functional theory (DFT) to confirm their relative formation energy (Δ*E*_rel_) and iteratively used to refine the generator. The second loop updates property predictors by sampling out-of-distribution (OOD), rare, and uncertain generative samples to improve accuracy. Arrows indicate the direction of data flow. *W* denotes the Wasserstein distance between material distributions, *H*_form_ refers to formation enthalpy, *ρ* and *u* represent density and uncertainty, respectively. Detailed sampling steps and formulated functions are provided in the Method section. (b) AL-driven continuous chemical spaces exploration. The blue circles represent generative exploration where the model samples and explores new chemical spaces. The arrow highlights the directions of the AL-driven continuous exploration. (c) Comparison of the distribution between known materials (gray curve) and the true distribution (red curve). The AL-driven distribution alignment helps correct biases in the generated data by aligning it with the true distribution (red line) while adjusting for bias in existing data.

Figure 1a illustrates our DuALGen framework for 2D materials discovery, consisting of two interlinked AL loops. These loops iteratively refine both the generative model and property predictors, driving continuous, unbounded exploration of 2D material space through data and model enhancement.

The goal of the generative AL loop is to guide the model in exploring a broader chemical space. As shown in Fig. 1b, the generative model’s exploration is often limited to regions near known data. DuALGen addresses this by introducing (1) novelty sampling, which allows the model to step outside the original data distribution; (2) diversity sampling, which encourages the model to explore in multiple directions; and (3) stability sampling, which focuses the model on discovering stable materials thus improving efficiency. It begins with a crystal diffusion variational autoencoder (CDVAE [21]) trained on 4535 stable 2D materials from the C2DB (2024 version), and sampling generated materials by three key metrics. (1) Stability: a formation enthalpy (*H*_form_) predictor estimates the relative formation energy (Δ*E*_rel_) to identify thermodynamically stable candidates. (2) Novelty: a modified structure matcher is used to quantify novelty, which dynamically compares lattice angles, lengths, and atomic sites to assess structural differences, enhancing conventional methods that only evaluate structural equivalence [22]. (3) Diversity: to address the bias and incompleteness in existing materials data, a diversity-driven metric samples materials with significant distributional differences to known compounds, thus expanding chemical space coverage. Selected candidates undergo density functional theory (DFT) validation for stability, after which they are added to the training dataset, enriching the data diversity and driving the exploration of previously uncharted material regions.

During the exploration of new chemical spaces, as shown in Fig. 1c, the distribution of known materials is often too localized to adequately cover new design space. This distributional gap, also referred to as data drift, leads to inaccurate property evaluations. To address this, we introduce a second AL loop that samples materials outside the known distribution, correcting the data distribution and maintaining predictive accuracy, which in turn enhances the stability assessment within the generative loop. This loop prioritizes three high-impact material types. (1) Out-of-distribution (OOD) materials: these materials differ significantly from known compounds. To identify them, we calculate the external density, which measures the concentration of known samples around a generated one; lower density indicates a larger distribution difference. (2) Rare materials: to reduce data redundancy, we define internal density as the local density of generated structures. Materials with low internal density are considered rare and selected to enhance diversity and sampling efficiency. (3) High-uncertainty materials: to overcome model uncertainty, we computed ensemble uncertainty for each prediction. Materials with high uncertainty are sampled to correct model bias and improve robustness. These materials are also validated via DFT and added back into the generative training set. Additionally, this loop also enhances other property predictors, such as the band gap regressor and metal/non-metal classifier, enabling robust identification of high-performance candidates, as discussed later.

### Active learning-enhanced generative novelty

**Figure 2.**
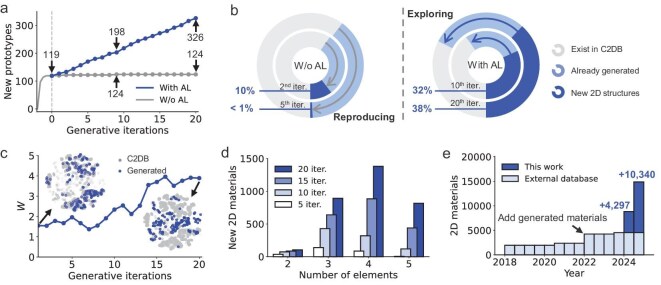
Continuous discovery of novel 2D materials via DuALGen. (a) New 2D structure prototypes in each generative iteration (10 000 materials per iteration), with and without AL. Materials with identical structures are counted as a single prototype to highlight the discovery of new structures. Details on prototype extraction and elemental substitution are provided in Fig. S2. (b) Distribution of generated structures in different generative iterations regarding to the novelty with and without AL. (c) Wasserstein distance (*W*) between generated materials and known materials at each sampling iteration. The inserts show the distribution of sampled materials (blue) and known data (gray) visualized by t-distributed stochastic neighbor embedding (t-SNE). (d) Distribution of materials with different number of elements during generative iterations. Details on elemental distribution is provided in a periodic table in Fig. S3. (e) Annual comparison of stable (Δ*E*_rel_ < 0.2 eV/atom) 2D materials in C2DB and generated by DuALGen.

Figure 2a demonstrates DuALGen’s ability for continuous discovery of novel 2D structures. Unlike standard generation models, where novelty diminishes rapidly after the first iteration (e.g. generating 10 000 structures yet discovering only 5 new prototypes), DuALGen consistently identifies ∼20 new structural prototypes per iteration. After 20 iterations, the system identifies 326 prototypes, tripling the output of baseline non-AL methods. As shown in Fig. 2b, although the generative model produces ∼40% novel materials in each generation, its exploration remains confined around known materials. If the model continues generating, it frequently repeats previously generated materials, causing rapid novelty decay. By the fifth generation, <1% of the materials are novel. However, with the introduction of active learning, each iteration incorporates more novel and diverse structures, maintaining >30% novelty in generation and enabling continuous exploration of chemical space.

To better demonstrate the impact of introducing new materials on the generative model, we plotted the distribution differences between the generated materials and known materials for each generation. As shown in Fig. 2c, the distribution difference steadily increases with each generation. From the latent space distribution, we can also observe that the generated materials gradually step into unexplored regions. From a compositional perspective, DuALGen also overcomes the data bias toward binary and ternary compounds in training data (Fig. 2d) achieving a 16-fold increase in quaternary materials and uncovers 816 quinary compounds over 20 iterations, effectively mapping previously unexplored regions. This iterative data enrichment enables DuALGen to transcend the limitations of training data, facilitating the continuous discovery of novel 2D materials. In practical application, DuALGen achieves a production rate of ∼1000 novel 2D materials monthly, a 30-fold acceleration over conventional methods, as illustrated by the time-dependent slope in Fig. 2e. This acceleration is scalable with additional computational resources, with further details provided in the Method section.

### Active learning-enhanced generative stability

**Figure 3.**
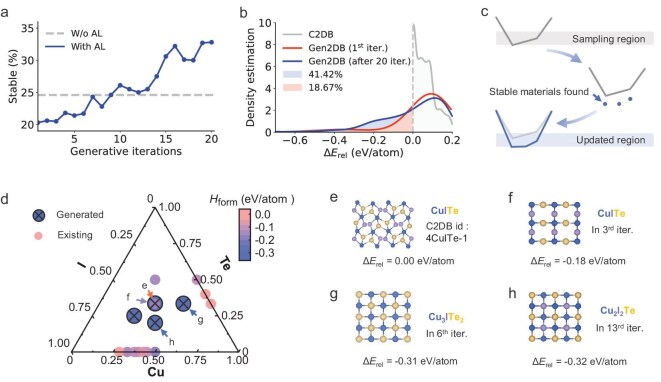
AL-enhanced generative models for discovering stable 2D materials. (a) Evolution of the rate of stable 2D materials identified through AL iterations. (b) Distribution of Δ*E*_rel_ comparing materials in the C2DB and those generated in the initial iteration and after 20 iterations. Negative Δ*E*_rel_ indicates higher stability relative to known stable references in C2DB. The probability density function is estimated via Gaussian kernel density estimation with optimized bandwidth. (c) Illustration of changes in the energy phase diagram during generative iterations, as well as the updated sampling range for stable materials. (d) Phase diagram for the Cu-I-Te system. Cross-marked dots denote materials generated by DuALGen, while small solid dots represent existing materials in C2DB. Color gradient from blue (most stable) to pink encodes Δ*E*_rel_ values. Crystal structures and Δ*E*_rel_ for selected materials are detailed in panels (e–h). (e) Crystal structure and unique identifier of the CuITe material with zero Δ*E*_rel_ in C2DB. (f–h) Novel stable configurations discovered by DuALGen, annotated with discovery iteration and corresponding Δ*E*_rel_ values.

To assess the impact of AL on generative stability, we systematically tracked the evolution of thermodynamically stable materials across AL iterations. Figure 3a shows a steady increase in stable material production, where the AL-enhanced generator outperforms the baseline model (without material updates) starting from the 10th iteration. This performance gap continues to widen in subsequent AL loops. A quantitative comparison in Fig. 3b reveals that after 20 iterations, 41.42% of materials generated by the AL-enhanced model exhibit a negative Δ*E*_rel_ (more stable than known materials in C2DB), compared to just 18.67% for the baseline generator. These results highlight DuALGen’s superior ability in improving the stability of generated materials.

Interestingly, a counterintuitive decline in stable outputs was observed during the early AL iterations, despite the reintroduction of DFT-confirmed stable materials. This phenomenon likely arises from the unreliable Δ*E*_rel_ evaluation caused by the inherent sparsity of known 2D materials, which are predominantly binary and ternary systems. As DuALGen explores higher-order multicomponent systems, the design space expands accordingly (Fig. 2d), leaving vast regions of the phase diagram unpopulated. In such sparse chemical spaces, the convex hull constructed from known materials may fail to represent the true thermodynamic ground states, leading to systematic overestimation of stability for generated candidates. The inclusion of such inaccurately assessed materials can temporarily degrade model performance, biasing it toward unstable configurations. Fortunately, this issue is self-correcting: as AL progresses, it uncovers increasingly stable and diverse materials, gradually populating the phase space and refining the convex hull as shown in Fig. 3c. This iterative enrichment improves the fidelity of stability assessments and enhances the robustness of the generative model.

The Cu-I-Te ternary system exemplifies the capability of DuALGen in discovering stable 2D materials. Figure 3d maps the known 2D materials, predominantly binary compounds clustered at the edges of the phase diagram, with only two ternary compounds identified. Figure 3e displays one ternary structure with zero Δ*E*_rel_. Our AL-enhanced generator identified four new ternary configurations (Fig. 3f–h), with a Δ*E*_rel_ of −0.18 eV/atom by the third generation, surpassing the stability of all known counterparts. Further iterations led to even more stable materials with the lowest Δ*E*_rel_ of −0.32 eV/atom achieved. Notably, this progress was made with only a few targeted DFT calculations for formation energy, demonstrating DuALGen’s exceptional efficiency in navigating complex composition spaces. In contrast to conventional crystal structure search methods [23,24], DuALGen substantially accelerates phase space exploration by concentrating computational resources on high-quality candidates guided by AL.

### Active learning-enhanced property prediction

**Figure 4.**
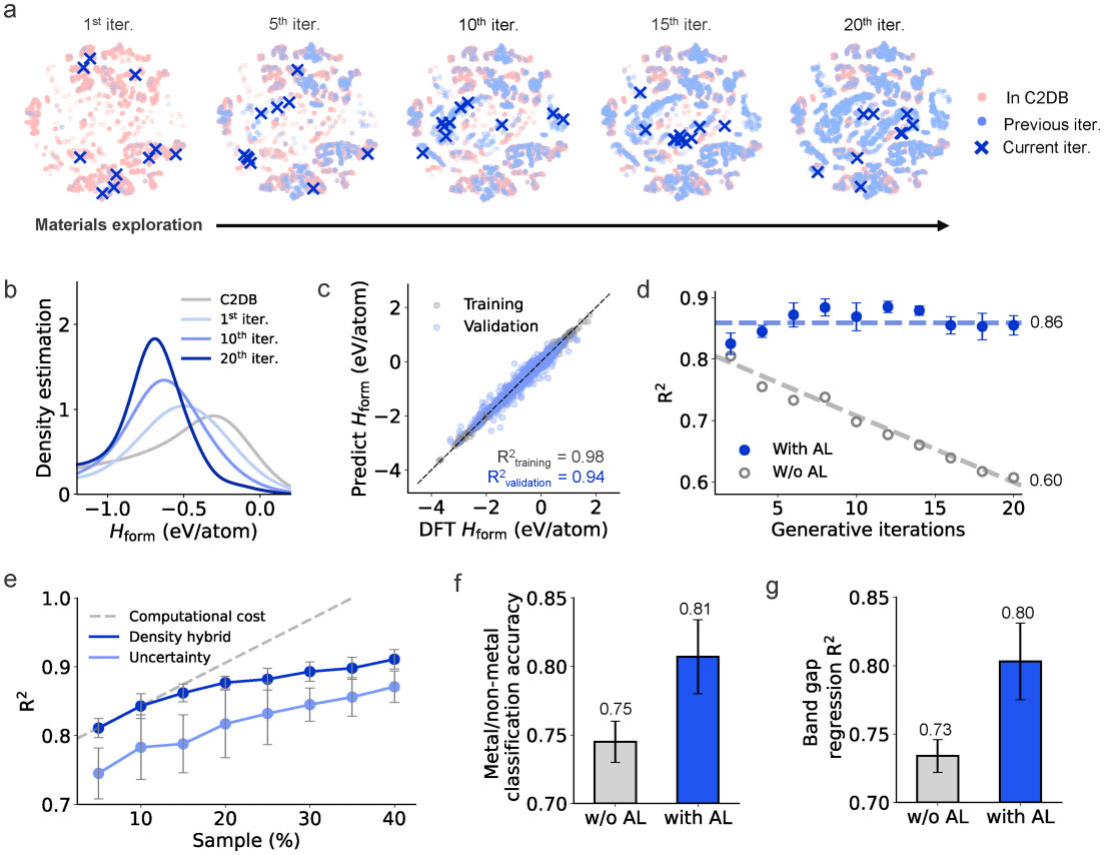
AL-driven mitigation of data drift and enhanced property prediction for generated materials. (a) Chemical space evolution. The t-distributed stochastic neighbor embedding (t-SNE) visualization of AL-iterated latent space (blue dots) expands progressively, compensating for underpopulated regions in the original C2DB dataset (pink dots). See Supplementary Movie for the dynamic exploration process. (b) *H*_form_ evolution across different AL iterations. The probability density function is estimated via Gaussian kernel density estimation with optimized bandwidth. (c) Prediction accuracy of *H*_form_ in C2DB. (d) Prediction accuracy of *H*_form_ predictors trained on C2DB for generated materials across different AL iterations, with and without AL. (e) Performance improvement as a function of sampling size under different metrics. Detailed performance improvements in each iteration and comparison of other uncertainty sampling methods are provided in Fig. S5a. AL-enhanced prediction accuracy on (f) classifying metal/non-metal and (g) band gap regression for generated materials.

The above analysis demonstrates that AL can significantly enhance generative ability, but this expanded exploration introduces new methodological challenges due to shifts in data distribution. Figure 4a maps the chemical landscape, where known materials form isolated clusters separated by vast unexplored regions. AL-driven sampling targets the boundaries of known materials and uncharted areas, guiding the generative model to explore a broader, more diverse space. After 20 iterations, the newly discovered materials fill the previously blank regions. However, this expansion shifts the data distribution over successive AL iterations, as evidenced by the varying patterns of blue and red dots. This drift is further reflected in the *H*_form_ distributions in Fig. 4b, where values progressively shift toward lower ranges. Such distributional drift poses a challenge to the reliability of property predictors.

Although the basic *H*_form_ prediction model achieves high accuracy, with a validation *R*^2^ of 0.94 on the C2DB (Fig. 4c), its performance deteriorates when applied to evaluating generated materials (Fig. 4d). This decline becomes more pronounced with successive AL iterations, underscoring a trade-off between exploration breadth and prediction reliability. To resolve this conflict, we propose a density-aware AL protocol that optimizes both material exploration and prediction reliability. In addition to conventional uncertainty sampling, our protocol introduces two complementary metrics: one calculates the local density of existing materials to target sparse regions in chemical space, enabling adaptive refinement of predictors for unexplored materials; the other evaluates the density of generated materials to identify rare candidates and minimize redundancy, thereby improving sampling efficiency. With this hybrid sampling protocol, the predictive model shows substantial performance recovery (Fig. 4d), with just 10% of samples maintaining accuracy >0.8. A systematic evaluation of sampling parameters (Fig. 4e) reveals two key insights. First, accuracy improvement gradually slows down, indicating diminishing returns for large-scale sampling. Second, density-based selection outperforms conventional uncertainty-based selection, especially at smaller sampling scales. These findings suggest a smaller sampling scale, combined with a density-based sampling function, offers an optimal balance between accuracy and efficiency.

In fact, the deterioration of model performance due to data shifts is quite common. We tested various generative architectures (including diffusion [21], flow-matching [25] and large language models [26] in Fig. S6) confirm that such performance degradation consistently occurs regardless of the specific generative model used. From the property perspective, the classification accuracy for metal/semiconductor properties decreases by 13.8% and the *R*^2^ for band gap prediction drops by 16.1% when applied to generated materials as shown in Fig. S4. However, the integration of AL maintains accuracy above 0.8 for both properties (Fig. 4f and g), effectively mitigating the impact of data shift. These results underscore the risks of using existing models on generated materials and demonstrate how AL can counteract these challenges. Beyond data shift mitigation, Gen2DB also establishes a new benchmark for assessing model extrapolation, a critical capability for discovering novel materials. Unlike conventional hold-out approaches that resample existing data [27], our platform generates genuinely out-of-distribution samples, enabling a more rigorous assessment of extrapolation performance. As shown in Fig. S7, comparison with traditional OOD test methods, such as leave-one-cluster-out validation (LOCOV), and Gen2DB, reveals that LOCOV overestimates extrapolation capability by 27% compared to Gen2DB.

### Functional materials in Gen2DB

Leveraging the DuALGen-enhanced property predictors, we have obtained >10 000 stable materials, including a batch of high-performance semiconductors, all archived in the open-access generated 2D materials database (Gen2DB). To ensure the chemical validity of the database, we excluded materials containing radioactive elements or those failing to meet charge neutrality, removing only 441 materials as illustrated in Fig. S3. Through three representative case studies, we highlight the key differences between Gen2DB materials and conventional counterparts, showcasing how the AL-enhanced generative model transcends the limitations of existing material databases.

**Figure 5.**
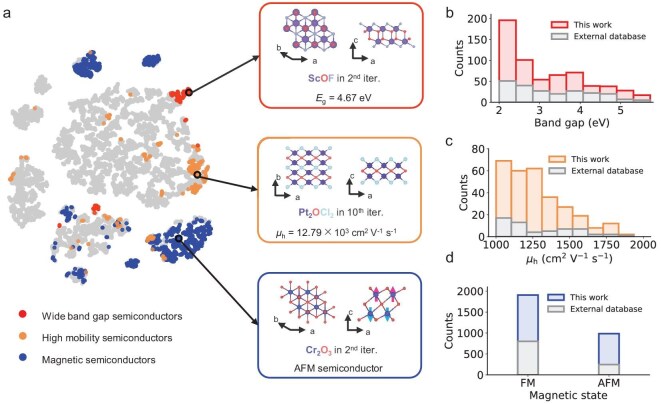
Functional 2D materials discovered in generated 2D materials database (Gen2DB). (a) t-SNE visualization of Gen2DB. We highlight three types of functional 2D semiconductors: intrinsic magnetism (blue), high hole mobility (orange), and wide band gap (red). Each type is represented by an example material highlighted in black circles with their crystal structures shown in the adjacent squares. The electronic structures and phonon dispersions of selected cases are provided in Fig. S8. The distribution of (b) band gap, (c) carrier mobility, and (d) magnetic ground state of 2D semiconductors in Gen2DB compared to external database C2DB. More details on property distribution are given in Fig. S9.

The development of 2D materials has traditionally centered on elemental monolayers (e.g. graphene [28], black phosphorus [29]) and transition metal dichalcogenides (e.g. MoS_2_ [30]). Differently, we present three novel 2D materials with unprecedented functional properties (Fig. 5a). The *p-3m1* ScOF features an ultra-wide band gap of 4.67 eV and unique structural characteristics including a seven-coordinated Sc center, deviating from the typical six-coordination observed in Sc-based compounds. This hypercoordinated configuration may lead to enhanced electronic complexity and unexpected properties [31]. Another promising material, *pccm* Pt_2_OCl_2_, exhibits exceptional high hole mobility (12.79 × 10^3^ cm^2^ V⁻^1^ s⁻^1^) and a direct 0.7 eV band gap, a combination rarely seen in established 2D semiconductors, making it a strong *p*-type candidate. Additionally, *p-3m1* Cr_2_O_3_ displays antiferromagnetic ordering, unlike conventional ferromagnetic Cr-based materials (e.g. CrI_3_ [32], Cr_2_Ge_2_Te_6_ [33]). This unique behavior arises from the near –180° Cr-O-Cr bonding between two Cr layers, enhancing super-exchange interactions and strengthening antiferromagnetic coupling (Fig. S10).

Experimental advancements support the synthetic feasibility of these materials. For instance, a structural analog of ScOF, layered ZrNCl, has already been synthesized [34] and a similar bulk ScOF exists, suggesting large potential for the synthesis of 2D ScOF. Additionally, 2D Cr_2_O_3_ has been successfully synthesized [35] and shares the same hexagonal unit cell as our predicted structure. While Pt_2_OCl_2_ presents greater structural uniqueness, the synthesis of 2D platinum oxides, such as PtO_2_ [36], suggests feasible pathways for its experimental realization. Beyond these three materials, Gen2DB houses thousands of high-performance candidates with critical functional properties, including wide band gap (Fig. 5b), high-mobility (Fig. 5c) and intrinsic magnetism (Fig. 5d), far surpassing existing databases. These findings not only showcase the superior properties of the generated materials but also demonstrate the immense potential of Gen2DB as a platform for ongoing innovation in functional 2D materials.

## DISCUSSION

This study presents DuALGen, a dual AL-enhanced framework for the iterative discovery of novel, diverse, and stable 2D materials through efficient sampling. By addressing the limitations of existing material datasets, i.e. small scale and limited diversity, DuALGen facilitates continuous exploration of previously uncharted materials design space. We also introduce a density-based sampling strategy to integrate AL into property prediction models, effectively mitigating the performance degradation caused by data drift between generated and known materials. Such AL-enhanced predictors demonstrate versatility across various functional properties. Moreover, we have launched Gen2DB, a growing database containing >10 000 novel and stable 2D materials, including thousands of high-performance candidates. Serving as a sustainable resource, Gen2DB accelerates material innovation and supports ongoing exploration of the vast material landscape. Our work provides both an expanding database and a self-driven, closed-loop strategy for materials discovery, advancing the field of 2D materials research.

The modular architecture of DuALGen enables broad generalizability and versatility, extending beyond 2D material generation. As a data-centric framework, DuALGen decouples model training from data sampling, allowing seamless integration of diverse generative and predictive models (such as flow matching [25] and large language models [26] as shown in Fig. S6) for various material systems. Furthermore, by incorporating conditional constraints (e.g. target properties or compositions) and integrating advanced optimization strategies such as Bayesian Optimization (BO) or Reinforcement Learning (RL) into the sampling loop, DuALGen can be extended into a powerful inverse design engine or a guided phase diagram explorer. While current results show no significant bias in terms of elemental composition and crystal structure, the risk of mode collapse remains when the generative model is retrained on self-generated data [37]. Addressing this challenge will require the development of more comprehensive evaluation metrics to identify high-value, low-probability candidates. Additionally, experimental synthesis [38,39] should be incorporated as the ultimate validation for the generated materials, ensuring that they are not only theoretically feasible but also practically viable for real-world applications.

## METHODS

### Sampling functions for generative models

To evaluate the stability, novelty and diversity of generated materials, we employed a three-step process. For stability, we defined a relative formation energy (Δ*E*_rel_) as a more stringent criterion than the standard convex hull energy (*E*_hull_). Δ*E*_rel_ compares the *H*_form_ of a generated material to the minimum *H*_form_ of all C2DB phases within the same chemical space (including subsets). This approach ensures that a candidate is not only stable at its specific stoichiometry but also competitive against alternative phases within the broader chemical space of its constituent elements. A threshold of Δ*E*_rel_ < 0.2 eV/atom (initially relaxed to 0.3 eV/atom) was used to identify stable candidates. For novelty, we calculated the structural novelty score (*S*_N_) using the StructureMatcher in pymatgen [22]. By framing the mismatch of lattice lengths (*L*), lattice angles (*A*) and atomic sites (*S*) as a Pareto optimization problem, *S*_N_ represents the shortest Euclidean distance from a structural equivalence boundary to the origin. *S*_N_ outperformed standard displacement metrics in identifying stable, high-symmetry structures as shown in Fig. S11:


\begin{eqnarray*}
{S}_N = \textit{argmin}\ \left| {\left| {L,\ A,\ S} \right|} \right|\!.
\end{eqnarray*}

From 1000 filtered candidates, 100 were selected by maximizing the Wasserstein distance (*W*) between the feature distributions (computed via Matminer [40]) of generated and known materials:


\begin{eqnarray*}
W\left( {E,G} \right) = min\mathop \sum \limits_{i = 1}^m \mathop \sum \limits_{j = 1}^n {f}_{ij}{| {| {{e}_i - {g}_j} |} |}_2,
\end{eqnarray*}

where *E* and *G* represent feature sets of known and generated materials, respectively, and *e* and *g* denote individual vectors. We further optimized for elemental diversity by evaluating the standard deviation of elemental distributions. The final set of 100 materials, which balance stability, novelty, and diversity, are then validated through DFT calculations.

### Sampling functions for predictive models

To refine property predictions, we utilized a distributive density (*ρ*) strategy based on the *k* = 5 nearest neighbors in the latent space. The density *ρ* is then calculated as:


\begin{eqnarray*}
\rho = \frac{1}{{k \cdot d{{\left( {x,{x}_k} \right)}}^2}}\mathop \sum \limits_{i = 1}^k \frac{1}{{d\left( {x,{x}_i} \right)}}.
\end{eqnarray*}

This metric captures the local material density in the high-dimensional feature space. A detailed example is provided in Fig. S12. We prioritized materials with low external density (*ρ*_E_), distinct from known materials, and low internal density (*ρ*_I_), rare among generated samples. Additionally, XGBoost [41] ensemble uncertainty was also introduced to sample regions of less confidence, such a hybrid approach significantly outperforms random sampling (Fig. S13).

### High-throughput DFT validations

Energy calculations were performed in two stages to balance accuracy and efficiency. First, a coarse optimization was conducted (EDIFFG = −0.2, *k*-mesh density = 0.05, fixed vacuum space = 15 Å) to discard unstable structures. Subsequently, high-precision optimization is performed under tighter criteria (EDIFFG = −0.01, *k*-mesh density = 0.03) to obtain the final energy. All reference materials from C2DB were recalculated under identical settings for consistency. Carrier mobility was estimated via transfer learning [42] and the deformation potential approximation [43]. Band gaps were computed using automated k-paths via VASPKIT [44,45], with SOC included for magnetic elements. Phonon spectra were obtained through a finite displacement method via the PHONOPY [46] code. All DFT calculations were performed using the Perdew-Burke-Ernzerhof [47] functional to balance accuracy and computational cost. More computational details including time cost and hyperparameter optimization are provided in Figs S14 and S15.

## Supplementary Material

nwag101_Supplemental_Files

## Data Availability

The training data for 2D materials used in this study were obtained from the C2DB database [12,13]. All generated 2D materials that have been validated to be thermodynamically stable through DFT calculations have been compiled into the Gen2DB dataset. This includes crystallographic structure files (in CIF format) and their corresponding DFT-calculated *H*_form_. This dataset is hosted within the source code repository provided below.

## CODE AVAILABILITY

All code for the DuALGen framework, including sampling strategies, generative workflows, and high-throughput DFT automation scripts, is openly available at https://github.com/XinYu-Chen98/DuALGen-Exploring-novel-2D-materials-with-generative-model-and-active-learning.
