# Supplementary material for: Continuous discovery of novel 2D materials via dual active learning-driven generative models
Source: Natl Sci Rev. 2026 Feb 12;13(7):nwag101. doi: 10.1093/nsr/nwag101 (PMC13107964; doi:10.1093/nsr/nwag101)
Supplement: nwag101_Supplemental_Files [file nwag101_supplemental_files.zip › Supplementary data.pdf]

Supplementary Information for

Continuous discovery of novel 2D materials via dual active learning-driven generative models

Xinyu Chen[1], Zhilong Song[2], Shuaihua Lu[1], Qian Chen[1], Yuanqiu Mo[3], Qionghua Zhou[1,2,*] and Jinlan Wang[1,2,*]

[1]Key Laboratory of Quantum Materials and Devices of Ministry of Education, School of Physics, Southeast University, Nanjing 211189, China;

[2]Suzhou Laboratory, Suzhou 215004, China;

[3]Jiangsu Provincial Key Laboratory of Networked Collective Intelligence, School of Mathematics, Southeast University, Nanjing 211189, China

***Corresponding authors**. E-mails: qh.zhou@seu.edu.cn; jlwang@seu.edu.cn

Table of Contents:

1. Data-induced Generative Mode Collapse. S2
2. Structure Prototype Extraction. S4
3. Elemental Distribution. S5
4. Predictive Model Degradation on Generated Materials. S7
5. Gen2DB as an Extrapolative Test Set. S10
6. Details of Generated Functional Materials. S12
7. Examples of AL Sampling Functions. S19
8. Optimizations on High-throughput DFT Calculations. S23

1 Data-induced Generative Mode Collapse.

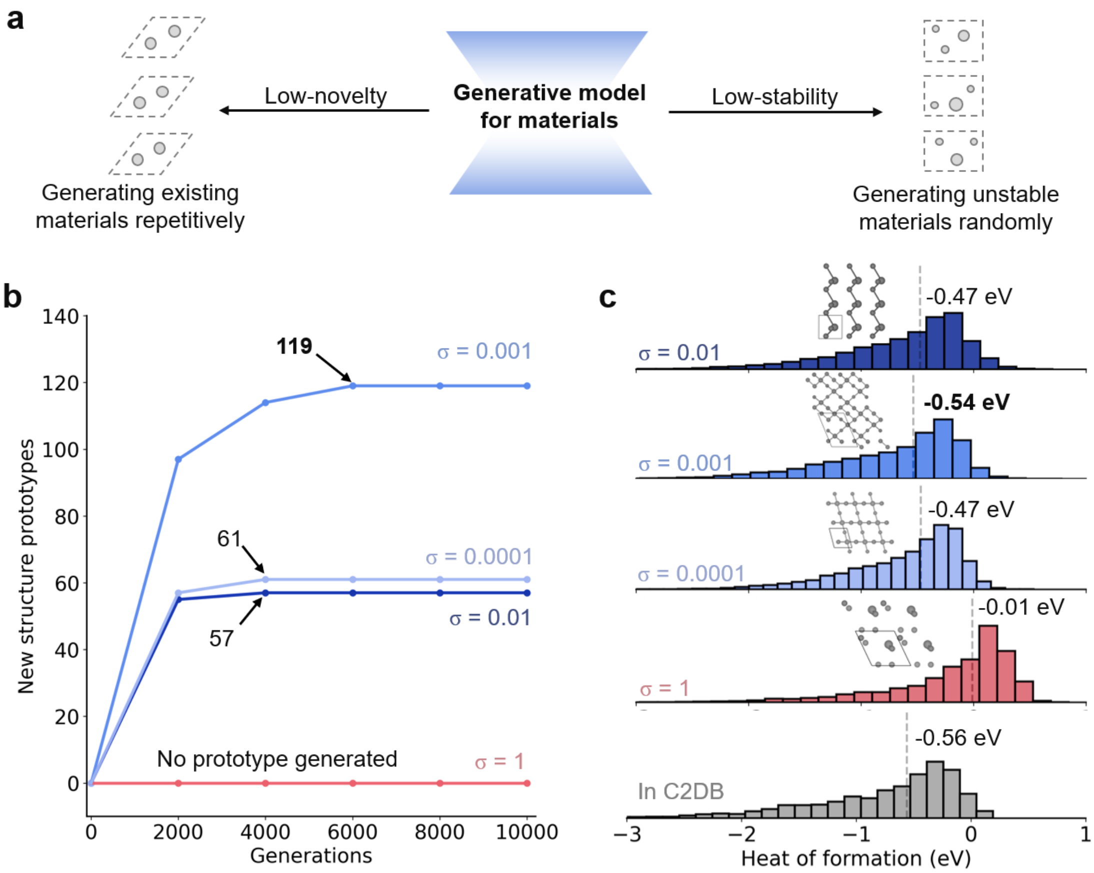

Figure S1. Mode collapses in materials generative model. (**a**) Two typical types of mode collapse: repetitive generation of known materials and uncontrolled generation of thermodynamically unstable structures. (**b**) Novelty of generated materials under different diffusion noise levels (σ), reflecting exploration ability. (**c**) Thermodynamic stability (heat of formation) of generated materials under varying σ. Insets display representative structures corresponding to the median heat of formation.

Figure S1a illustrates two common failure modes in materials generation: the repetitive generation of structures resembling known materials, and the random production of thermodynamically unstable configurations. A persistent decline in novelty is observed over multiple generation cycles, where most generated samples begin to converge toward previously seen or trivial structures. This phenomenon remains even when adjusting key generative parameters such as the diffusion noise level (σ), as shown in Fig. S1b, indicating that the exploration capacity of generative model is fundamentally limited. This degradation primarily stems from a constrained latent space biased toward the training distribution. The model, being trained exclusively on known materials, lacks the capacity to explore regions beyond that

empirical domain. As a result, the scale and diversity of the training dataset play a critical role in defining the generative frontier.

Meanwhile, the stability of generated materials exhibits significant sensitivity to the diffusion process. As shown in Fig. S1c, increasing the noise level (e.g., $\sigma = 1$) results in highly disordered, metastable structures. In contrast, smaller σ values yield more ordered and stable materials. However, regardless of σ, the heat of formation (H_{form}) distribution of generated samples closely mirrors that of the training set, typically following a long-tail profile: most materials cluster in high-energy, unstable regions, while low-energy, stable candidates remain sparse. This intrinsic bias leads to two critical limitations: (I) Generated materials often exhibit poor thermodynamic stability, making them impractical for experimental realization. (II) The energy prediction model becomes dominated by high-energy samples, reducing its reliability in the low-energy regime, which is the region of primary interest for discovering synthesizable, stable materials. These findings highlight the importance of both training data diversity and generative robustness in overcoming distributional collapse and achieving practically meaningful materials discovery.

The modular architecture of DuALGen, which prioritizes Active Learning (AL) over Bayesian Optimization (BO) or Reinforcement Learning (RL) for material discovery, is motivated by three primary considerations: First, unlike the "exploitation-heavy" nature of BO and RL which often traps sampling in narrow, high-performing regions, our AL strategy emphasizes unbiased exploration to resolve historical data biases and map the entire 2D structural landscape. Second, AL is significantly more sample-efficient for high-cost DFT landscapes; while RL requires vast feedback episodes to converge, AL maximizes "information gain" from a minimal set of validated samples, accelerating the discovery of stable prototypes. Finally, AL offers superior model-agnostic flexibility, allowing for the seamless integration of diverse generative architectures without the complex redesign of policy gradients or system-specific reward functions, thereby ensuring the framework's scalability and robustness.

2 Structure Prototype Extraction.

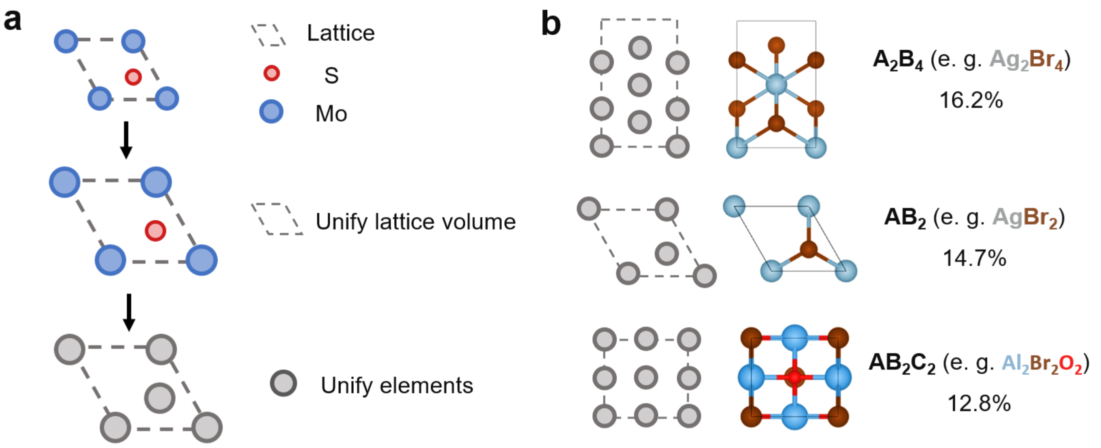

Figure S2. Structure prototype extraction and element replacement. (**a**) An example of prototype extraction from MoS_2, including lattice normalization and element unification. (**b**) The top three structural prototypes in C2DB alongside representative materials.

In generative materials discovery, we prioritize the generation of novel structure prototypes, followed by the systematic expansion of chemical space through elemental substitution. This two-step strategy offers a more efficient exploration compared to relying solely on generative models to directly produce such materials. As shown in Fig. S2a, to decouple structural motifs from elemental identities, we first normalize the unit cell and standardize all atomic species. For 2D systems, we also fix the vacuum space to 15 Å to eliminate interlayer interactions. To avoid incorporating randomly generated, low-quality configurations, we define a structure prototype only if the motif appears more than five times in the dataset.

Figure S2b highlights the top three structural prototypes within the C2DB, along with representative examples. Notably, these three prototypes alone account for 43.7% of the entire database, underscoring the presence of a highly skewed structural distribution. The construction of 2D materials databases heavily relies on element substitution, which accelerates database expansion, but also results in a concentration of materials around a small set of structural backbones, ultimately limiting the generative diversity and novelty.

3 Elemental Distribution.

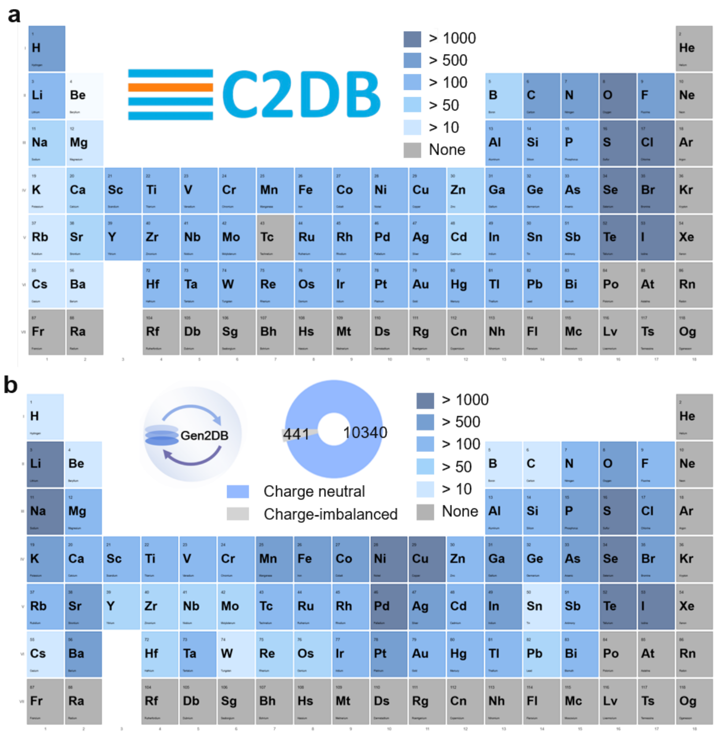

Figure S3. Elemental distribution in existing and generated materials. (**a**) Elemental occurrence in the C2DB database. (**b**) Elemental occurrence in the generated material set. Lanthanides and actinides are excluded; color intensity reflects the frequency of element appearance.

The elemental distribution in Fig. S3a reveals a distinct pattern in C2DB: most transition metals appear with frequencies ranging from 100 to 500, while halogens and chalcogens often exceed 1,000 occurrences. This imbalance reflects the underlying data construction strategy, extensive elemental substitution within a limited set of structural prototypes. While efficient, this approach inherently biases the dataset toward well-known material families such as transition metal dichalcogenides (TMDs) and graphene derivatives.

However, these prototypes typically represent local optima in chemical space based on known experimental stability. Their substituted variants may lack similar thermodynamic favorability, leaving large regions of potentially more stable structures unexplored.

In contrast, the elemental distribution of generated materials in Fig. S3b indicates a shift in exploration. Elements such as Ni, Cu, and Li are generated with high

frequency, despite being relatively underrepresented in traditional databases. This suggests that the generative framework helps uncover novel compound-forming potential in previously under-sampled regions of the elemental space, highlighting its role in broadening discovery beyond the historical biases of curated datasets.

4 Predictive Model Degradation on Generated Materials

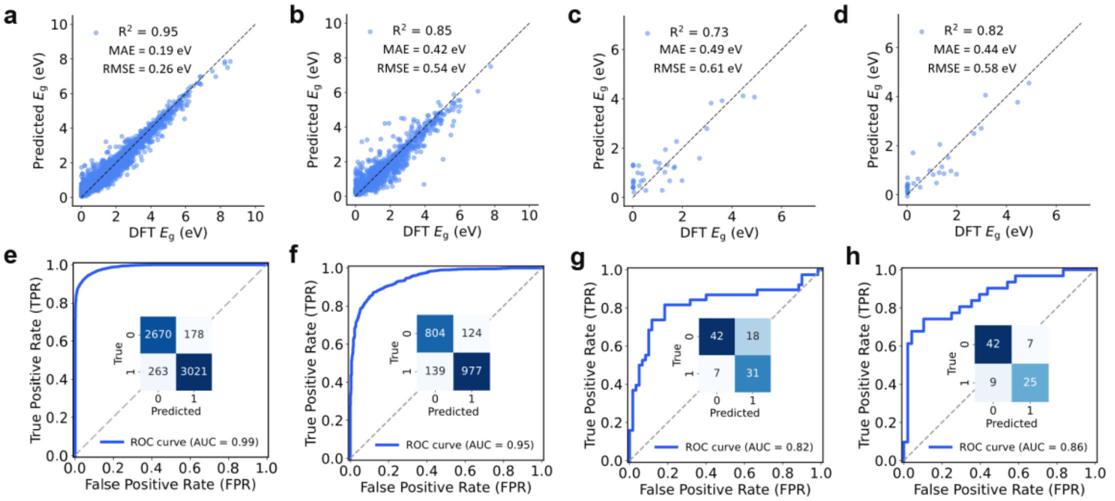

Figure S4. Model performance for band gap regression and metal/non-metal classification. Coefficient of determination (R^2) for the band gap regression model on the (**a**) training set, (**b**) validation set, (**c**) test set, and (**d**) test set after active learning (AL). Area under the ROC curve (AUC) for the metal/non-metal classification model on the (**e**) training set, (**f**) validation set, (**g**) test set, and (**h**) test set after AL. Insets show the corresponding confusion matrices.

To evaluate the degradation of property prediction models on generated materials, we consider two representative tasks: band gap regression and metal/non-metal classification.

As shown in Fig. S4a-b, the band gap regression model trained on C2DB semiconductors achieves high accuracy, with $R^2 > 0.85$ on both training and validation sets. However, performance drops significantly on 100 randomly selected generated samples (Fig. S4c), with R^2 falling to 0.73. After including 10% of the generative samples via density- and uncertainty-based sampling, the model's performance on the remaining generated data improves markedly ($R^2 = 0.82$, Fig. S4d), nearly recovering its original accuracy.

A similar pattern is observed in the classification task. The metal/non-metal classifier achieves AUC > 0.90 on C2DB data (Fig. S4e-f), but drops to AUC = 0.82 with 25 misclassifications on generative samples (Fig. S4g). Incorporating 10% of sampled generative data raises the AUC to 0.86, reducing misclassifications to 16 (Fig. S4h).

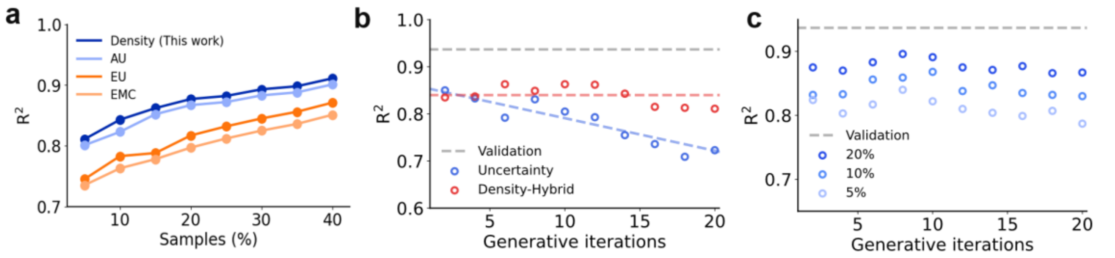

Figure S5. Active learning-enhanced property prediction across generative iterations. (**a**) R^2 scores for formation energy with different sampling rate, comparing the original density-based sampling (This work) with ensemble uncertainty (EU) expected model change (EMC) and adversarial uncertainty (AU) strategies. The blue-shaded lines (Density and AU) represent data uncertainty sampling, while the orange-shaded lines represent model uncertainty-based sampling (**b**) R^2 scores for formation energy prediction at different sampling rates. (**c**) R^2 scores using different sampling strategies for active learning.

In Fig. 4e, we demonstrated the overall impact of different sampling ratios and strategies on the predictive performance for generated materials.

To validate such scheme, we benchmarked the density-based sampling against more representative model-uncertainty (e.g., Expected Model Change, EMC) and data-uncertainty (e.g., Adversarial Uncertainty, AU) strategies. As illustrated in Fig. S5a, the density-based approach consistently yields the most robust performance gains, confirming that representative coverage of the structural manifold is paramount for building reliable global models of 2D materials. Notably, data-centric metrics generally outperform model-centric ones in our framework. This superiority stems from the fact that DuALGen primarily addresses an Out-of-Distribution (OOD) problem; in regions far from historical data, model-internal uncertainty is often poorly calibrated, whereas data-centric metrics directly target the distribution shift, more effectively correcting the inherent bias of the training set.

We further analyze the effect of active learning at each generative iteration. Each iteration involves 10,000 generated materials, among which we evaluate the top 100 candidates selected based on stability, novelty and diversity. It is worth noting that earlier generative iterations produce materials that are more similar in distribution to the known materials, while later iterations tend to generate materials that deviate more significantly from the known distribution.

As shown in Fig. S5a, increasing the sampling ratio consistently improves the

predictive accuracy across all generative iterations, regardless of their distance from the training distribution. However, in Fig. S5b, we observe that uncertainty-based sampling performs well only in the early stages. Its effectiveness declines as the generative steps progress. This is because model uncertainty is determined solely based on the training data and model capacity, as the distributional gap between generated and known materials widens in later iterations, uncertainty estimates derived from the original data become less reliable. In contrast, incorporating density-based sampling, which explicitly accounts for distributional differences between known and generated materials, mitigates this issue. It consistently improves predictive performance across all iterations.

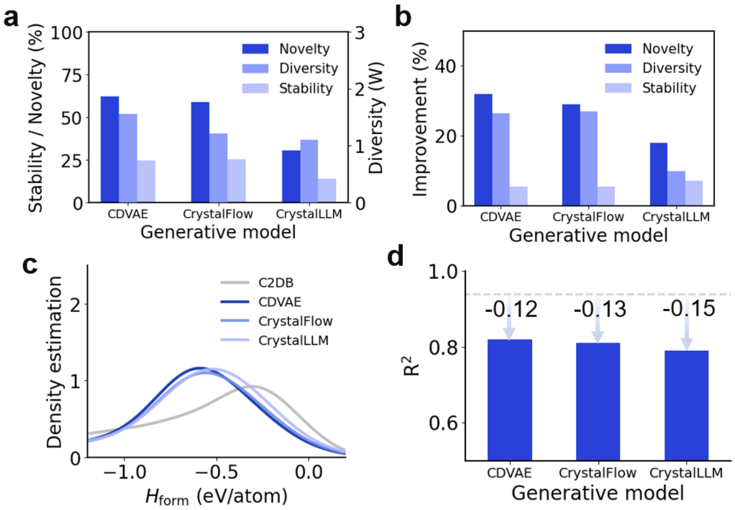

Figure S6. Universality of data drift across different generative architectures. (**a**) Comparison of novelty and stability among CDVAE, CrystalFlow, and CrystalLLM. (**b**) Improvement of novelty, diversity and stability after 5 active learning iterations for different models. (**c**) Distribution of formation energy (H_{form}) for materials generated by different models, showing distinct deviations from the original database distribution. (**d**) Degradation in predictor performance (R^2) caused by the distribution shift.

We evaluated CDVAE against CrystalFlow (flow-matching) and CrystalLLM (Transformer-based) to assess backbone influence on discovery efficiency (Fig. S6a). GNN-based diffusion and flow models (CDVAE and CrystalFlow) outperformed CrystalLLM in novelty and stability, as graph-based representations more effectively capture the lattice symmetries and atomic environments of 2D materials than text-string formats. GANs were excluded due to their inherent training instability and limited structural exploration capabilities. Crucially, all architectures exhibited consistent performance gains after several active learning iterations within DuALGen (Fig. S6b). This universal improvement confirms that the framework effectively refines the structural manifold regardless of the generative engine, ensuring high compatibility with current and future state-of-the-art architectures.

5 Gen2DB as an Extrapolative Test Set.

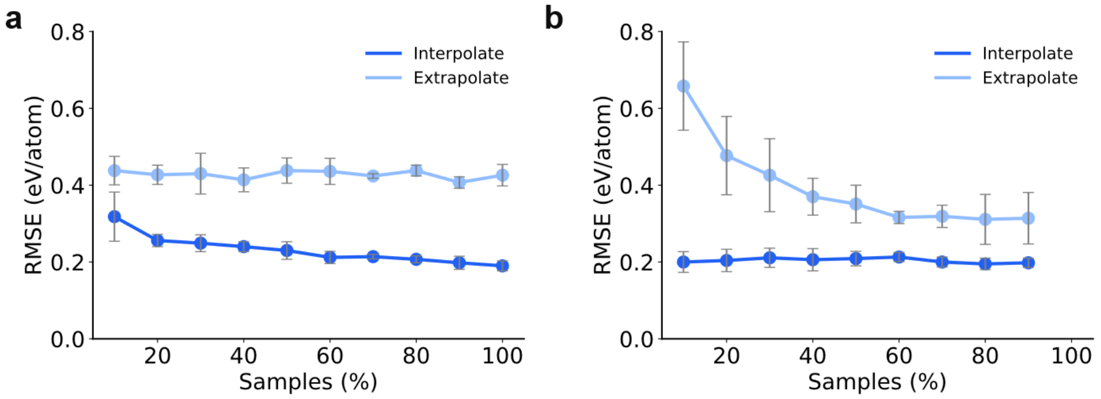

Figure S7. Extrapolative testing using (a) Gen2DB and (b) Leave-One-Cluster-Out Validation (LOCOV). The interpolation set corresponds to the validation set in C2DB. In Gen2DB, the entire generative database is used as the extrapolation set. In contrast, LOCOV defines the extrapolation set by holding out a subset of C2DB.

Given the significant distributional shift and much larger scale of Gen2DB compared to known materials, it is well suited for extrapolative evaluation. Gen2DB reflects more realistic out-of-distribution (OOD) scenarios and enables robust, data-insensitive assessments. Here, we demonstrate its advantages over conventional hold-out validation strategies [1].

As shown in Fig. S7a, models trained with increasing amounts of formation energy data exhibit steady performance improvement on the validation set (interpolation) of known materials, with a gradual reduction in RMSE. However, their performance on Gen2DB remains consistently poor. To compare with traditional hold-out approaches, we clustered the known materials into 10 groups and sequentially held out one to nine clusters as extrapolation set, with the remaining used for training (corresponding to training samples from 10% to 90% in Fig. S7b). We observed that while extrapolative performance improves with more training data, the validation performance does not show the same trend, in fact, it initially worsens before improving. This counterintuitive behavior illustrates the complex relationship between training diversity and extrapolation performance. When only one cluster is used for training, the materials are relatively homogeneous, resulting in better validation accuracy despite the small training size. However, the model performs significantly worse on the extrapolation set (the remaining nine clusters), with the

RMSE over three times higher than that on the validation set. As the number of training clusters increases, extrapolative performance improves, which can be attributed to both increased training diversity and reduced extrapolation diversity.

This analysis reveals two major limitations of conventional hold-out validation for extrapolation testing. First, the size and diversity of the held-out set are typically much smaller than those of the training set, which is inconsistent with real-world materials discovery scenarios where the known materials represent only a small fraction of the full chemical space. This can lead to an overestimation of extrapolation ability. Second, holding out data inevitably reduces the quantity and diversity of the training set, which may also impair extrapolative performance. In contrast, Gen2DB offers a much larger and more diverse set of materials beyond the known data, enabling more robust and realistic extrapolation benchmarking.

6 Details of Generated Functional Materials.

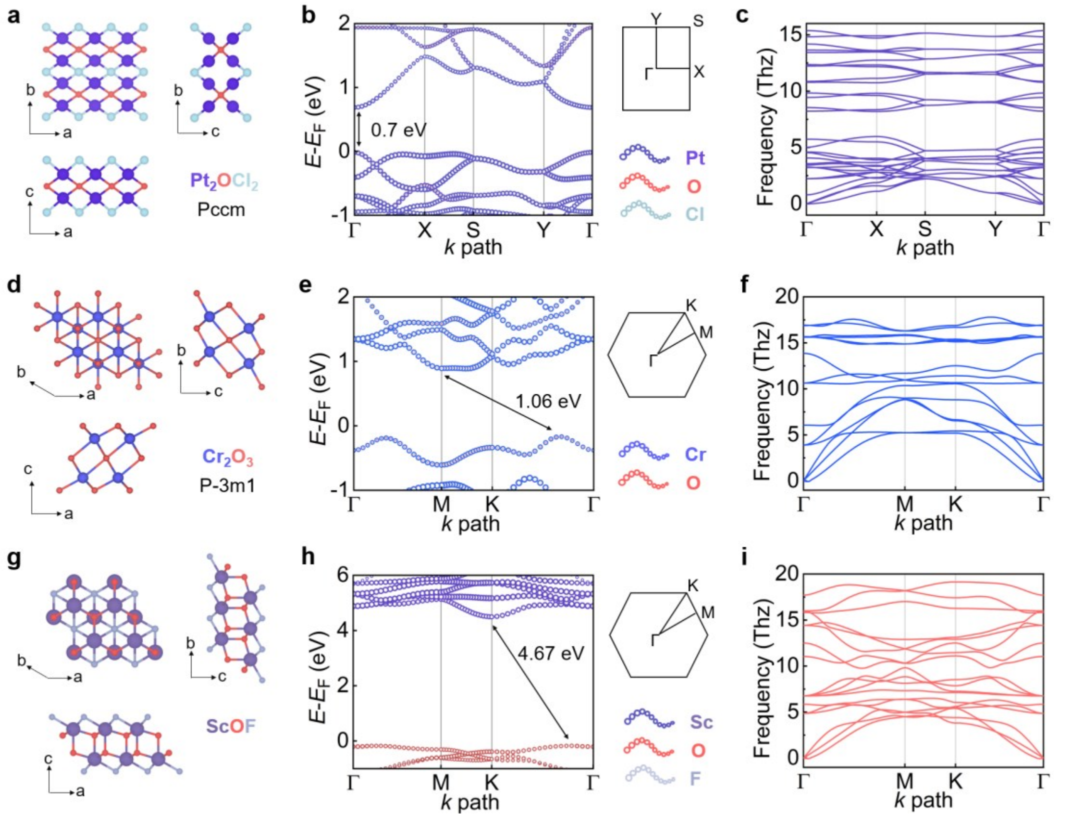

Figure S8. Electronic structure and dynamical stability of representative functional materials identified in Figure 5. (**a**) Crystal structure, (**b**) electronic structure and (**c**) phonon spectrum of high mobility Pt_2OCl_2. (**d**) Crystal structure, (**e**) electronic structure and (**f**) phonon spectrum of anti-ferromagnetic Crt_2O_3. (**g**) Crystal structure, (**h**) electronic structure and (**i**) phonon spectrum of wide band gap ScOF. The electronic structures are depicted through element-projected band structures, with illustrations of the k-path and contributions from different elements shown alongside.

Figure S8 presents the electronic structures and dynamical stabilities of three representative generated materials highlighted in Fig. 5. Pt_2OCl_2 exhibits a direct band gap of 0.7 eV, while Cr_2O_3 possesses an indirect band gap of 1.06 eV, which is comparable to that of silicon, suggesting strong potential for semiconductor applications. In contrast, ScOF shows an ultra-wide band gap of 4.67 eV, significantly exceeding that of conventional wide-band-gap materials such as GaN [2], indicating promising applications in high-power electronic devices. All three materials exhibit phonon spectra free of significant imaginary frequencies, confirming their dynamical stability. In addition, their thermodynamic stability has been validated based on E_{hull}, supporting their potential synthesizability.

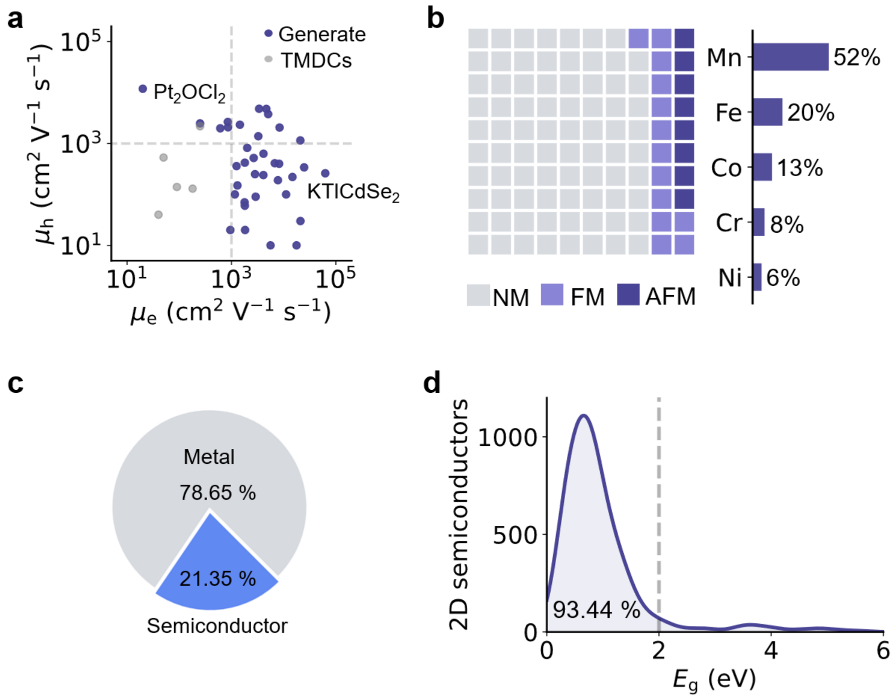

Figure S9. Distribution of generated functional materials. (**a**) Distribution of high-mobility materials with electron or hole mobility exceeding 10^3 cm^2 V^{-1} s^{-1}. Materials with highest electron and hole mobility are highlighted. (**b**) Distribution of magnetic materials, along with the five most frequently occurring magnetic elements. (**c**) Distribution of metallic and semiconducting materials. (**d**) Distribution of semiconductors by band gap.

Figure S8 presents the overall distribution of functional materials generated by the model. The distribution of high-mobility materials in Fig. S9a shows that most candidates exhibit high electron mobility, while materials with high hole mobility are relatively rare. Nevertheless, the mobilities of these materials significantly exceed those of commonly used 2D TMDs [3]. Figure S9b illustrates the distribution of magnetic materials in Gen2DB. Approximately 20% of the generated materials exhibit magnetism, with ferromagnetic phases being the most prevalent and antiferromagnetic phases comparatively less common. Among these magnetic materials, manganese (Mn) is the dominant magnetic element, present in over 50% of the cases. Figures S9c-d show that the majority of materials in Gen2DB are metallic. Among the semiconductors, 93.44% have a band gap smaller than 2 eV, indicating that wide-band-gap materials are relatively scarce. From this dataset, we identified and validated 53 wide band gap materials (> 2 eV), as listed in Table S1, and 37 high-mobility materials (> 10^3 cm^2 V^{-1} s^{-1}.), as shown in Supplementary Table 2.

Table S1. Summary of validated wide band gap materials. The material ID prefix indicates the active learning iteration in which the material was generated, and whether it is a prototype or derived through elemental replacement (ER).

Material ID	Formula	Space group	E_{fermi} (eV)	E_g (eV)	Band character
R12ER-111	$LiMg_2Cl_5$	Pmm2	-5.00	4.47	Indirect
R12-19	$NaMg_2Cl_5$	Pmm2	-4.69	4.17	Indirect
R2ER-22	ScOCl	P-3m1	-3.30	3.33	Indirect
R2ER-39	Mg_2SeI_2	P-3m1	-1.83	2.60	Indirect
R2ER-41	Sr_2SeI_2	P-3m1	-2.61	3.11	Indirect
R2ER-50	As_2O_3	P1	-4.15	3.98	Indirect
R2ER-65	ScOF	P-3m1	-4.64	4.67	Indirect
R2ER-87	ZrO_2	P-3m1	-4.77	4.45	Indirect
R2-11	Ca_2SI_2	P-3m1	-2.10	2.75	Indirect
R2-17	ScBrO	P-3m1	-2.51	2.63	Indirect
R2-1	BiF_3	Pm	-5.00	3.16	Indirect
R2-31	HfO_2	P-3m1	-5.00	4.90	Indirect
R3ER-0	AlSBr	P1	-1.17	2.42	Indirect
R3ER-76	$Mg_3Se_2I_2$	P3m1	-1.01	2.55	Indirect
R3ER-77	$Sr_3Se_2I_2$	P3m1	-1.66	2.73	Indirect
R6ER-113	Ba_2I_2O	P-4m2	-2.50	2.07	Indirect
R6ER-313	Ca_2Cl_2O	P-4m2	-3.70	3.25	Indirect
R6ER-315	Ba_2Br_2O	P-4m2	-2.99	2.13	Indirect
R6ER-317	Mg_2Br_2O	P-4m2	-2.80	2.08	Indirect
R6ER-318	Sr_2Br_2O	P-4m2	-3.39	2.63	Indirect
R6ER-319	Ca_2SBr_2	P-4m2	-2.23	2.33	Indirect
R6ER-398	Li_2MgS_2	P-4m2	-1.01	2.05	Indirect
R6ER-405	Li_2BeSe_2	P-4m2	-1.27	2.30	Indirect
R6ER-43	$BaSrCl_2O$	Pm	-3.67	2.52	Indirect
R6ER-50	$BaCaBr_2O$	Pm	-3.49	2.15	Indirect
R6ER-65	Sr_2Cl_2O	P-4m2	-3.39	2.75	Indirect
R6ER-71	Sr_2SI_2	P-4m2	-2.20	2.49	Indirect
R6ER-81	Sr_2SeI_2	P-4m2	-1.76	2.12	Indirect
R6-19	Ca_2Br_2O	P-4m2	-3.53	2.90	Indirect
R6-1	$BaSrBr_2O$	Pm	-3.60	2.34	Direct
R6-25	Li_2BeS_2	P1	-1.01	2.44	Indirect
R6-29	Sr_2I_2O	P-4m2	-2.51	2.20	Indirect
R7ER-14	Sr_2Cl_2O	Pm	-3.94	2.91	Indirect
R7ER-198	NaBr	P4/mmm	-3.89	3.85	Indirect
R7ER-199	NaCl	P4/mmm	-4.26	4.55	Indirect

R7ER-200	NaI	P4/mmm	-2.94	3.40	Indirect
R7ER-25	$ZrOF_2$	P1	-5.00	3.75	Indirect
R7ER-35	Mg_2Cl_2O	P1	-3.21	2.21	Indirect
R7ER-36	$BaSrCl_2O$	Pm	-3.70	2.78	Indirect
R7ER-45	$SrCaCl_2O$	Pm	-3.96	2.90	Direct
R7ER-47	Bi_2HBrO_3	P1	-1.71	2.71	Indirect
R7ER-6	$HfOF_2$	Pmm2	-6.64	3.92	Indirect
R7-2	Bi_2HClO_3	P1	-1.91	2.86	Indirect
R8ER-48	$MgBr_2$	P3m1	-3.03	4.63	Direct
R8ER-51	$CaBr_2$	P3m1	-3.41	4.76	Indirect
R8ER-56	BaI_2	P3m1	-2.37	3.82	Indirect
R8ER-78	$SrBr_2$	P3m1	-3.68	4.69	Indirect
R8ER-79	$BaCl_2$	P3m1	-4.23	5.22	Indirect
R8-0	$BaBr_2$	P3m1	-3.81	4.57	Indirect
R9ER-117	$HfSCl_2$	C2/m	-4.21	2.33	Indirect
R9ER-24	$ZrSF_2$	C2/m	-4.73	2.14	Indirect
R9ER-51	$HfOF_2$	C2/m	-6.98	5.88	Indirect
R9-16	$HfSF_2$	C2/m	-5.00	2.38	Indirect

Table S2. Summary of validated high mobility materials. All materials are converted to orthogonal lattices, which are labeled as x and y. Since the deformation potential approximation and effective mass approximation require first-order and second-order fitting, results with a fitness less than 0.9 may be unreliable thus are represented by '/'.

Material ID	Formula	E_g (eV)	carrier	E_x (eV)	E_y (eV)	m^*_x (m_0)	m^*_y (m_0)	μ_x (10^3 cm^2/V·s)	μ_y
R2-12	Ge_2TeI_2	1.43	e	5.40	7.05	0.17	0.17	0.95	0.84
			h	4.96	5.15	48.58	0.48	0.00	0.02
R2ER-107	As_2Te_3	0.78	e	/	1.07	0.33	0.39	/	4.65
			h	2.69	6.69	0.12	0.11	4.78	1.35
R6ER-66	InSI	1.38	e	2.67	2.33	0.35	0.35	0.91	1.16
			h	3.58	4.12	/	0.34	/	/
R6ER-96	BTeI	0.29	e	1.48	/	0.55	0.27	2.00	/
			h	2.09	1.96	0.55	0.55	0.71	0.82
R6ER-99	GaTeI	0.79	e	/	1.87	0.15	0.30	/	2.99
			h	0.99	1.09	13.27	13.27	0.00	0.00
R6ER-113	Ba_2I_2O	2.07	e	1.46	1.46	0.51	0.51	1.76	1.76
			h	3.61	3.61	1.01	1.01	0.07	0.07
R6ER-115	InSeI	1.43	e	1.10	2.60	0.23	0.24	8.17	1.58
			h	1.75	0.70	0.24	15.3	0.40	0.04
R7ER-35	Mg_2Cl_2O	2.21	e	1.40	1.16	0.58	0.55	3.69	4.08
			h	1.67	5.97	3.18	0.41	0.24	0.10
R8-7	GaTeI	0.20	e	0.41	4.84	1.33	0.91	3.38	0.03
			h	0.25	3.80	2.10	0.91	4.81	0.04
R10ER-1	Pt_2SeBr_2	0.83	e	0.59	3.81	0.98	0.93	1.25	0.18
			h	1.24	1.52	0.33	14.84	0.36	0.03
R10ER-82	Pt_2SeF_2	0.48	e	0.29	2.75	0.89	0.52	11.12	0.76
			h	1.49	3.71	14.35	0.52	0.01	0.10
R10ER-103	Pt_2OCl_2	0.70	e	2.26	8.33	1.09	27.01	0.02	0.00
			h	0.40	0.76	1.09	0.52	4.73	11.88
R11ER-82	In_2Te_4Pb	0.53	e	6.62	6.68	0.16	0.13	1.29	1.49
			h	0.57	0.44	7.76	1.01	0.18	2.34
R24ER-95	$CdAl_2Se_4$	0.78	e	8.20	8.15	0.15	0.20	1.30	1.02
			h	4.79	4.54	0.61	2.10	0.05	0.15
R24ER-98	$CdGa_2Se_4$	0.07	e	7.63	7.32	0.12	0.10	2.58	4.10
			h	5.27	4.23	1.19	0.33	0.09	0.63
R24ER-136	CdSe	0.87	e	/	2.66	0.10	0.09	/	17.59
			h	5.20	4.07	12.59	1.26	0.00	0.01
R24ER-139	Cd_3Te_2S	0.37	e	2.98	2.30	0.10	0.15	11.40	14.68

			h	3.02	3.25	0.29	13.30	0.22	0.01
R25ER-27	$CaGa_2Se_4$	0.14	e	6.96	7.42	0.12	0.13	2.82	2.54
			h	4.53	4.48	26.37	0.20	0.00	0.25
R25ER-139	Tl_2HgSe_2	0.52	e	3.68	3.68	0.16	0.16	2.66	2.66
			h	2.87	2.87	0.59	0.59	0.52	0.52
R26-24	CdSe	0.22	e	2.30	2.37	0.11	0.12	20.77	17.33
			h	4.27	4.21	0.16	0.96	1.15	0.20
R26ER-317	Mg_2Br_2O	2.09	e	3.69	3.69	0.33	0.33	1.80	1.80
			h	6.54	6.54	1.66	1.66	0.06	0.06
R26ER-318	Sr_2Br_2O	2.62	e	0.71	0.71	1.24	1.24	1.82	1.82
			h	3.87	3.87	2.26	2.26	0.02	0.02
R26ER-319	Ca_2Br_2S	2.32	e	0.43	0.43	0.62	0.62	19.46	19.46
			h	3.36	3.36	/	/	/	/
R26ER-394	ZnSe	0.62	e	5.59	3.98	0.19	0.16	2.22	5.04
			h	4.08	5.05	0.38	0.12	1.77	3.78
R26ER-562	$KTlCdSe_2$	1.16	e	0.43	6.97	0.35	0.13	62.53	0.40
			h	3.15	1.86	1.14	0.66	0.09	0.26
R26ER-571	$KTlZnSe_2$	1.10	e	1.71	/	0.28	0.14	/	2.75
			h	/	1.75	1.11	0.62	/	0.41
R29-19	$CaIn_2Te_4$	0.11	e	6.37	6.39	0.12	0.10	2.61	2.89
			h	3.75	4.42	18.25	0.31	0.00	0.09
R29ER-316	$LiAsSe_2$	0.33	e	/	/	0.06	0.07	/	/
			h	5.28	5.28	0.06	0.06	8.84	8.84
R29ER-318	$LiGaSe_2$	0.58	e	2.30	2.29	0.63	0.63	0.85	0.86
			h	2.27	2.27	0.25	0.25	2.08	2.08
R29ER-320	$LiInSe_2$	0.46	e	5.58	5.58	0.24	0.24	0.85	0.85
			h	1.74	1.74	0.24	0.24	2.65	2.65
R29ER-424	$CaIn_2Se_4$	0.35	e	5.70	5.70	0.12	0.12	2.86	2.86
			h	3.61	3.80	1.07	1.07	1.39	1.39
R30ER-406	$BaZnS_2$	0.93	e	0.87	0.86	0.21	0.39	0.25	0.21
			h	1.28	/	0.68	0.26	2.47	/
R30ER-879	Na_2ZnS_2	2.16	e	0.60	0.60	0.58	0.58	7.75	7.75
			h	2.11	2.11	/	/	/	/
R30ER-881	Li_2HgS_2	0.81	e	2.80	2.80	0.24	0.24	1.80	1.80
			h	3.90	3.90	/	/	/	/
R30ER-886	Na_2HgS_2	1.61	e	0.52	0.52	0.36	0.36	24.63	24.63
			h	2.16	2.16	/	/	/	/
R30ER-1090	$LiAlTe_2$	0.38	e	0.84	0.84	0.55	0.55	8.16	8.32
			h	3.22	3.22	/	/	/	/

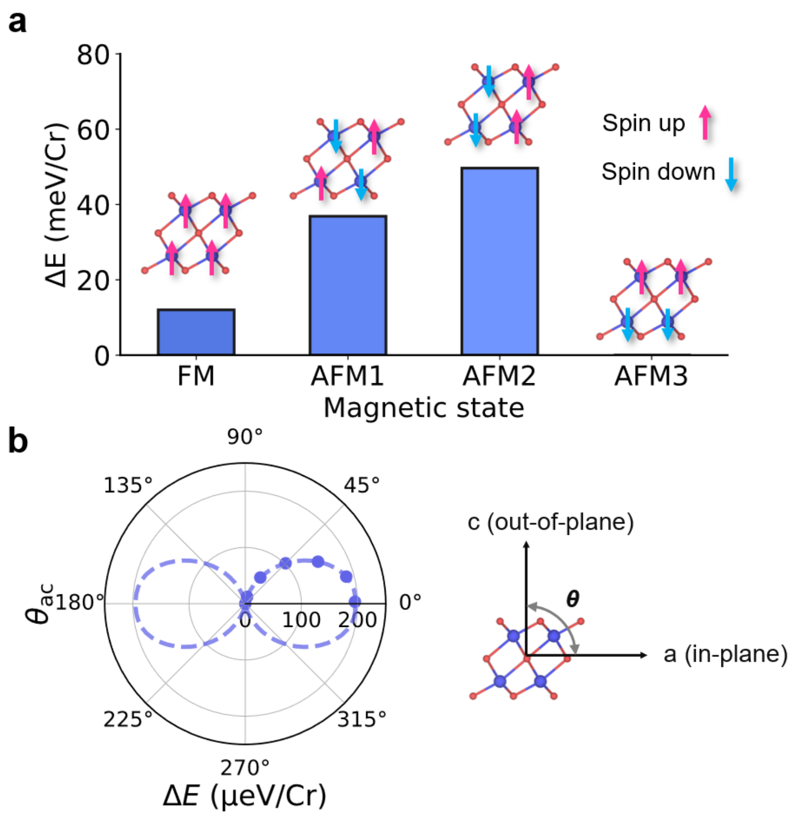

Figure S10. The magnetic ground state and anisotropy of Cr_2O_3. (**a**) Relative energies of different magnetic configurations with respect to the ground state. Insets show side views of the corresponding spin arrangements. (**b**) Magnetic anisotropy energy as a function of the magnetization axis direction, where θ denotes the angle from in-plane ($\theta = 0°$) to out-of-plane ($\theta = 90°$).

Figure S10a shows the relative energies of various magnetic configurations of Cr_2O_3, identifying the AFM3 phase as the magnetic ground state. In this configuration, Cr atoms exhibit antiferromagnetic coupling between two sublayers, while within each sublayer, the Cr atoms are ferromagnetically aligned. Figure S10b presents the magnetic anisotropy energy, which reaches 193 μeV/Cr, comparable to that of commonly studied Cr-based magnetic materials such as CrI_3 [4]. Notably, unlike many layered magnets with in-plane easy axes, Cr_2O_3 exhibits an out-of-plane easy axis, suggesting its potential for achieving higher storage densities in spintronic applications.

7 Examples of AL Sampling Functions.

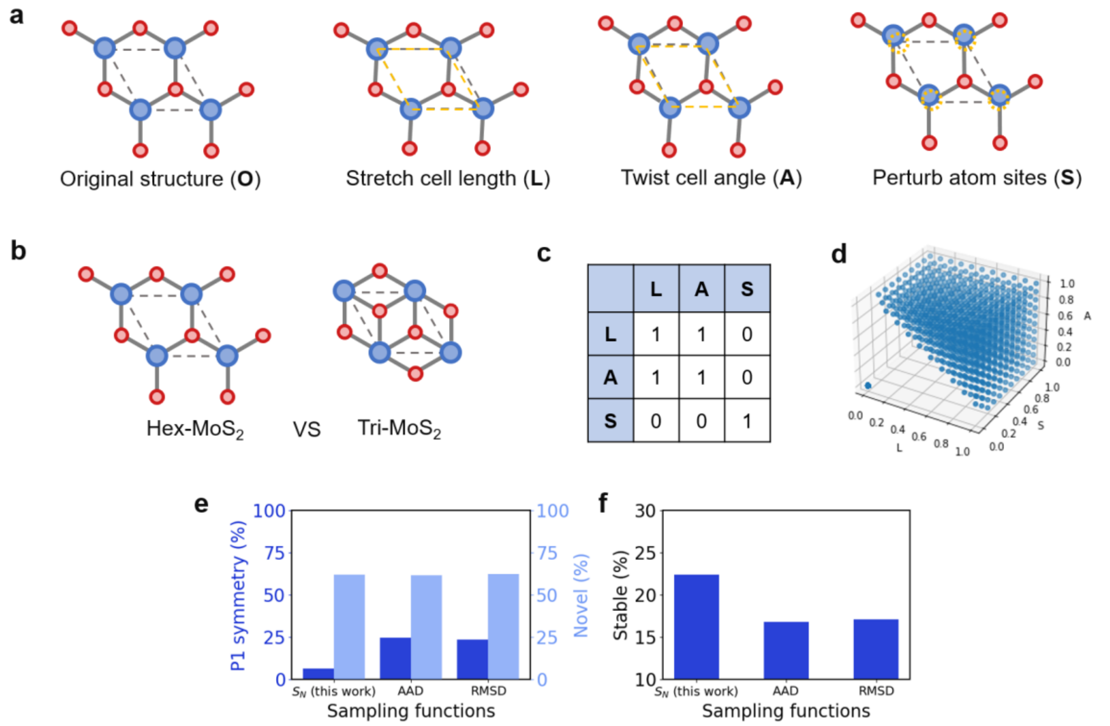

Figure S11. The illustration of structural novelty quantification. (**a**) Schematic representation of the original structure (O) and three types of structural perturbations: stretched lattice length (L), twisted lattice angle (A), and perturbed atomic sites (S). (**b**) An example comparing structural differences between hexagonal and trigonal phases of MoS_2. (**c**) Qualitative similarity matrix used for structural comparison, where a value of 1 indicates that two structures share the same structural feature under a given criterion, and 0 indicates a difference. In this example, the hexagonal and trigonal MoS_2 phases share similar lattice lengths and angles, but differ in atomic site positions. (**d**) Three-dimensional plot of the critical criteria for L, S, and A. The blue region represents the combinations of (L, A, S) thresholds under which two structures are considered equivalent. (**e**) Comparison of material novelty and symmetry achieved using the SN score versus traditional metrics like average atomic displacement (AAD) and root-mean-square deviation (RMSD). (**f**) The corresponding success rate in discovering stable materials.

Figure S11 provides a concrete example illustrating how structural novelty can be quantitatively assessed. In Fig. S11a, we show the original configuration of a crystal structure along with three representative types of structural deformation: stretched lattice length (L), twisted lattice angle (A), and perturbed atomic sites (S).

Traditional structure matching methods, such as the StructureMatcher in pymatgen [5], determine structural similarity based on fixed threshold values—for instance, the default settings are:

$$L_{th} = 0.2, A_{th} = 5°, S_{th} = 0.4\ \text{Å} \qquad eq.1$$

where L_{th} is the maximum allowed relative difference in lattice length, A_{th} is the allowed deviation in lattice angles, and S_{th} is the maximum displacement allowed for atomic sites.

While this approach is useful for qualitative structure matching (i.e., determining if two structures are "the same" or "different"), it does not offer a quantitative measure of how different two structures are when the match fails. To overcome this, we propose a method that generalizes the threshold parameters into a continuous search space. Let the structural difference between two structures be defined as a point in the 3D threshold space:

$$T = (L, A, S) \qquad eq. 2$$

We define a match indicator function $f(T)$, where:

$$f(T) = \begin{cases} 1 & \text{if the structures match under thresholds } T \\ 0 & \text{otherwise} \end{cases} \qquad eq. 3$$

As shown in Figs. S11b-c, we use the hexagonal and trigonal phases of MoS_2 as an example. Our algorithm searches over the normalized threshold space $T \in [0,1]^3$, where: L = 0 means the lattice lengths must match exactly; L = 1 allows up to the maximum lattice constant of either structure. A = 0 requires identical lattice angles; A = 1 corresponds to an angular deviation of up to 180°. S = 0 requires atomic sites to be in perfect alignment; S = 1 allows displacements up to the maximum interatomic distance.

We construct a match region $M = \{T \mid f(T) = 1\}$, and visualize this region in threshold space. As shown in Fig. S11d, the boundary of M forms a surface representing the critical thresholds beyond which the two structures begin to be recognized as different. Finally, we define the quantitative structural dissimilarity D between two structures as the minimum Euclidean distance from the origin to the boundary of the match region:

$$D = \min_{T \in M} \|T\|_2 \qquad eq. 4$$

This scalar value $D \in [0, \sqrt{3}]$ provides a quantitative measure of the degree of

structural novelty.

To validate the effectiveness of the S_N score, we performed a quantitative comparison against established local metrics, including average atomic displacement (AAD) and root-mean-square deviation (RMSD), within the generative sampling loop. As illustrated in Fig. S11e, while local metrics can enhance structural novelty by favoring large atomic deviations, they frequently result in highly distorted, low-symmetry geometries that are often unphysical. In contrast, our S_N score achieves a significantly higher success rate in discovering stable compounds (Fig. S11f) by balancing novelty with physical plausibility. Unlike local displacement metrics that act as surrogates for atomic jitters, S_N incorporates global lattice distortion and symmetry-breaking into its evaluation. By accounting for the overall crystal framework rather than isolated atomic positions, S_N effectively guides the generator toward structurally unique yet energetically stable 2D materials.

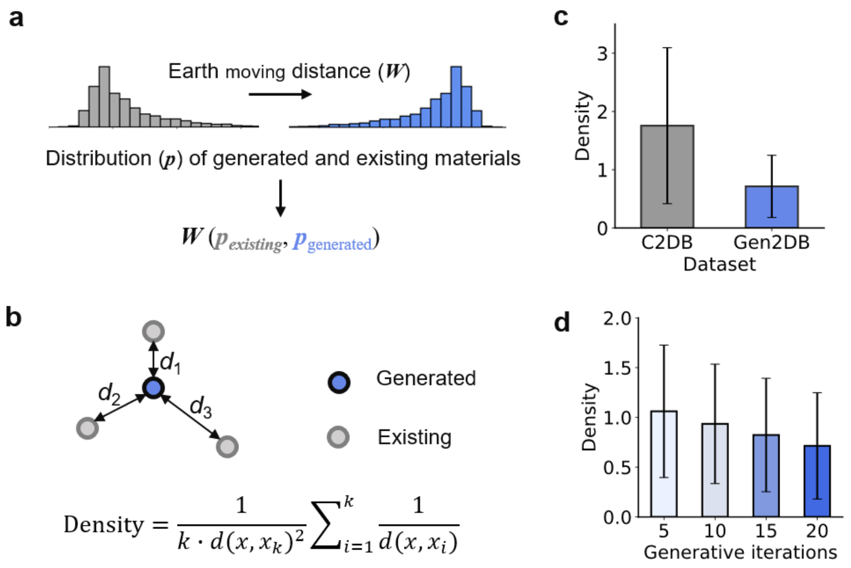

Figure S12. Quantifying distributional shifts and sample-level diversity in generated materials. (**a**) Schematic illustration of the Wasserstein distance (W), used to evaluate the global distributional difference between Gen2DB and C2DB. (**b**) Conceptual diagram of local density estimation in latent space. (**c**) Comparison of internal sample densities between C2DB and Gen2DB. (**d**) Evolution of sample density during active learning, reflecting increasing deviation from known data.

Figure S12a illustrates the computation of the Wasserstein distance (W), which evaluates the overall distributional difference between the generated materials and C2DB. Beyond global distributional shifts, we introduce a sample-level diversity

metric inspired by the *k*-nearest neighbor algorithm (Fig. S12b). For each generated sample, its local density in latent space is estimated based on distances to nearby points. Samples surrounded by few known materials are considered out-of-distribution (OOD), while those surrounded by few other generated samples are regarded as rare, contributing to diversity by reducing redundancy. Figure S12c, Gen2DB exhibits significantly lower internal density than C2DB, indicating a broader and more diverse material distribution. Figure S12d further shows a progressive density decline over active learning iterations, reflecting increasing deviation from known data. The large standard deviation suggests continued dispersion and thus sustained diversity.

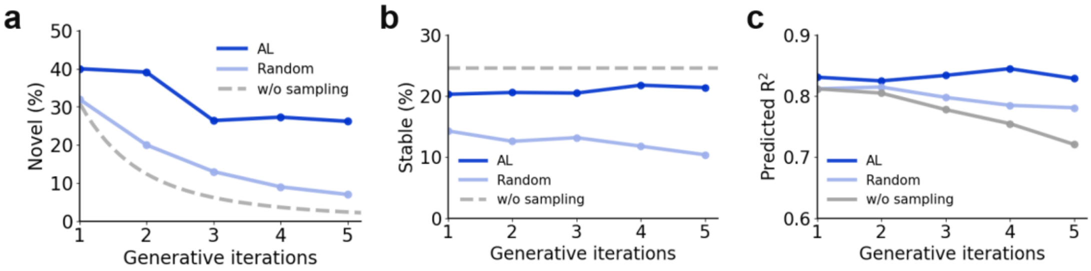

Figure S13. Comparison between active learning (AL) and random sampling. Evolution of (**a**) material novelty, and (**b**) structural stability for generated candidates. (**c**) Improvement in energy prediction accuracy (R^2) on generated materials.

8 Optimizations on High-throughput DFT Calculations.

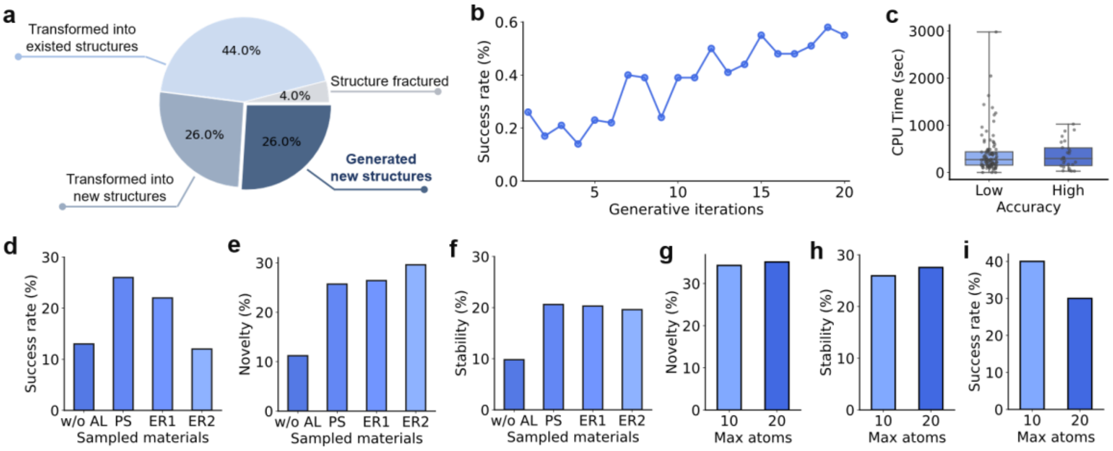

Figure S14. Optimization of generative parameters and validation efficiency. (**a**) Number of successful and failed calculations in the initial sampling round. (**b**) Success rate of structure generation and validation as a function of generative iteration. (**c**) Computational time required to validate 100 sampled materials. Comparison of (**d**) success rate, (**e**) novelty rate, and (**f**) stability rate of generated materials using different strategies: without active learning (w/o AL), sampling only prototype structures (PS), replacing one element in prototypes (ER1), and replacing two elements (ER2). Effect of atomic complexity on generation outcomes, showing (**g**) success rate, (H) novelty rate, and (I) stability rate for generated materials with a maximum atom count of 10 and 20.

As shown in Fig. S14a, not all sampled structures can be successfully validated, the initial generation round achieved a success rate of only 26%. The primary cause of failure stems from structural transformations during relaxation, where a significant portion of the generated structures collapsed into known ones. This indicates that many generated candidates were dynamically unstable. However, this issue was gradually alleviated as active learning improved the reliability of the stability predictor. As shown in Fig. S14b, the success rate exceeded 50% by the 20th generation. The time cost of the two-step structural optimization process in the first generation is shown in Fig. S14c. As the success rate improves with each iteration, the overall computational efficiency of the framework also increases.

The DuALGen framework applies functional constraints to ensure the quality of sampled candidates, but the sampling size is another critical hyperparameter. If too few structures are sampled, their influence compared to the original data may be negligible; conversely, if too many are sampled, metastable structures may undermine the stability of the generation process. We therefore designed three distinct sampling

strategies: Sampling 100 prototype structures (PS); Replacing one element per prototype (ER1, yielding ~400 candidates); Replacing two elements per prototype (ER2, yielding ~1500 candidates). As shown in Figs. S14d-f, increasing the sampling scale improves novelty, but multi-element replacement introduces a large number of unstable structures, which significantly reduces the validation success rate and impairs the overall stability of the generated materials. Based on this trade-off, we ultimately chose to replace only one element per iteration.

Additionally, since the majority of materials in C2DB have fewer than 10 atoms per unit cell, we evaluated the effect of atomic complexity by comparing generation with a maximum atom count of 10 and 20. As shown in Figs. S14g-i, generating larger 2D materials does not significantly impact novelty or stability, but it greatly reduces the success rate of validation. Consequently, we temporarily restrict the maximum atom count to 10. Notably, this constraint does not cause any significant drop in novelty, suggesting that the 2D structural space below 10 atoms remains largely unexplored. However, with recent advances in synthesis, high-entropy 2D materials and 2D material with more than 10 atoms per unit cell have been reported [6,7], and we plan to lift this restriction in the future to explore a broader material design space.

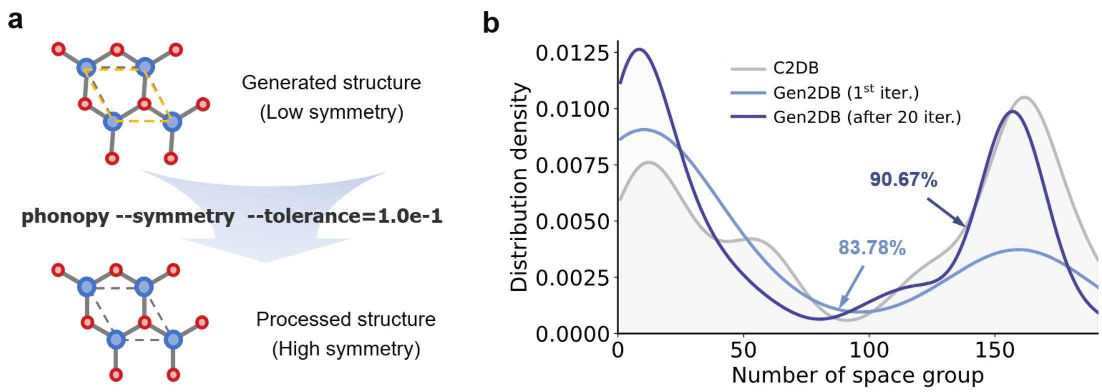

Figure S15. Evolution of symmetry in generated materials. (**a**) Preprocessing low-symmetry materials. (**b**) Distribution of space groups in C2DB and Gen2DB across different active learning generations. The arrow points out the percentage of non-P1 space group materials.

In diffusion-based generation, the presence of small perturbations often leads to atomic positions deviating from ideal symmetric sites. As a result, most generated materials are initially classified into the low-symmetry P1 space group when analyzed using conventional symmetry detection methods. To address this issue, we employed the symmetry-finding functionality in PHONONPY to recover the intrinsic crystallographic symmetry of each structure, as illustrated in Fig. S15a. After applying symmetry reconstruction, the majority of structures are no longer assigned to the P1 group. Moreover, as shown in Fig. S15b, the proportion of high-symmetry materials increases progressively with each active learning iteration, suggesting that the generative model becomes more capable of producing well-ordered, symmetric crystal structures over time.

References

1. Liao W, Yuan R, Xue X *et al.* Unsupervised learning-aided extrapolation for accelerated design of superalloys. *npj Comput Mater* 2024; **10**: 171.
2. He J, Cheng W, Wang Q *et al.* Recent Advances in GaN-Based Power HEMT Devices. *Adv Electron Mater* 2021; **7**: 2001045.
3. Cheng L, Zhang C, Liu Y. Why Two-Dimensional Semiconductors Generally Have Low Electron Mobility. *Phys Rev Lett* 2020; **125**: 177701.
4. Huang B, Clark G, Navarro-Moratalla E *et al.* Layer-dependent ferromagnetism in a van der Waals crystal down to the monolayer limit. *Nature* 2017; **546**: 270-3.
5. Ong SP, Richards WD, Jain A *et al.* Python Materials Genomics (pymatgen): A robust, open-source python library for materials analysis. *Comput Mater Sci* 2013; **68**: 314-9.
6. Zhu CY, Zhang MR, Chen Q *et al.* Magnesium niobate as a high-κ gate dielectric for two-dimensional electronics. *Nat Electron* 2024; **7**: 1137-46.
7. Mei H, Zhang Y, Zhang P *et al.* Entropy Engineering of 2D Materials. *Adv Sci* 2024; **11**: 2409404.
