# Supplementary material for: Continuous discovery of novel 2D materials via dual active learning-driven generative models
Source: Natl Sci Rev. 2026 Feb 12;13(7):nwag101. doi: 10.1093/nsr/nwag101 (PMC13107964; doi:10.1093/nsr/nwag101)
Supplement: nwag101_Supplemental_Files [file nwag101_supplemental_files.zip › supplementary_code.docx]

"""All source code used in this work is included in the present file.

Each code block is labeled with a header starting with

#### Code <number>

At the end of this file, we also provide the exact command-line inputs required to execute the corresponding codes.

"""

####code1: utils_ALsampling.py

import argparse

import os

import shutil

import numpy as np

import pandas as pd

from scipy.stats import wasserstein_distance

from pymatgen.core import Structure

from pymatgen.io.cif import CifParser

from pymatgen.io.vasp.inputs import Poscar

import ast

from collections import Counter

import random

from functionsG2DB import scale_vacuum_thickness

parser = argparse.ArgumentParser(description="Process some files.")

parser.add_argument('--source_fold', type=str, required=True, help="Path to the source folder")

parser.add_argument('--target_fold', type=str, required=True, help="Path to the target folder")

parser.add_argument('--gendata', type=str, required=True, help="Path to the generated data CSV file")

parser.add_argument('--gen_magpie', type=str, required=True, help="Path to the MAGPIE numpy file")

args = parser.parse_args()

source_fold = args.source_fold

target_fold = args.target_fold

gendata = args.gendata

gen_magpie = args.gen_magpie

# Test W-distance

data1 = np.load(gen_magpie)

data1 = np.where(np.isnan(data1), 0, data1)

print(len(data1))

data2 = np.load('MAGPIEC2DB.npy')

data2 = np.where(np.isnan(data2), 0, data2)

print(len(data2))

emd = wasserstein_distance(data1.flatten(), data2.flatten())

print('Wasserstein distance to C2DB:', emd)

def scale_list(lst):

min_val = min(lst)

max_val = max(lst)

return [(x - min_val) / (max_val - min_val) for x in lst]

# Scoring and sampling

data = pd.read_csv(gendata)

name = data['name'].values.tolist()

ehull = data['ehull'].values.tolist()

uniq = data['uniqueness'].values.tolist()

elem = data['elements'].values.tolist()

for i in range(len(elem)):

elem[i] = ast.literal_eval(elem[i])

#print(elem[i][0])

sub_names = []

EM_dist = []

ELE_div = []

stability = []

uniqueness = []

for rs in range(50):

data_filtered1 = data[data['uniqueness'] != 0]

data_filtered = data_filtered1[data_filtered1['ehull'] <= 0.3]

data_sorted = data_filtered.sort_values(by='ehull', ascending=True)

random.seed(rs)

print(len(data_sorted['name'].values.tolist()))

sub_name = random.sample(data_sorted['name'].values.tolist(), 150)

sub_names.append(sub_name)

sub_data1 = []

sub_elem = []

sub_s = 0

sub_u = 0

for i in range(len(sub_name)):

ids = name.index(sub_name[i])

sub_data1.append(data1[ids])

sub_elem += elem[ids]

sub_s += ehull[ids]

sub_u += uniq[ids]

element_counts = Counter(sub_elem)

frequency_list = list(element_counts.values())

#print(np.std(frequency_list))

ELE_div.append(np.std(frequency_list))

EM_dist.append(wasserstein_distance(np.array(sub_data1).flatten(), data2.flatten()))

#plot_t_SNE(sub_data1,data2)

print(wasserstein_distance(np.array(sub_data1).flatten(), data2.flatten()))

stability.append(sub_s)

uniqueness.append(sub_u)

#scaling

score_elediv = scale_list(ELE_div)

score_emdis = scale_list(EM_dist)

score_stable = scale_list(stability)

score_unique = scale_list(uniqueness)

sum_list = [a - b - c + d for a, b, c, d in zip(score_emdis, score_elediv,score_stable,score_unique)]

files_to_sample = sub_names[sum_list.index(max(sum_list))]

os.makedirs(target_fold, exist_ok=True)

for file_name in files_to_sample:

try:

source_file = os.path.join(source_fold, file_name)

destination_file = os.path.join(target_fold, file_name)

if os.path.exists(source_file):

try:

shutil.copyfile(source_file, destination_file)

#print(f' {file_name} copied to B.')

except Exception as e:

print(f'Error copy {file_name} : {e}')

else:

print(f'File {file_name} dose not exist in A.')

except:

print(file_name)

os.makedirs(os.path.join(target_fold, 'POSCARS'), exist_ok=True)

for file_name in os.listdir(target_fold):

try:

file = os.path.join(target_fold, file_name)

parser = CifParser(file)

structure = scale_vacuum_thickness(parser.get_structures(primitive=False)[0], 15)

poscar = Poscar(structure)

poscar.write_file(os.path.join(target_fold, "POSCARS", "POSCAR" + file_name.split('.')[0]))

print(file)

except:

print(file_name)

####code2: utils_ele_replace.py

import pandas as pd

import os

import shutil

from mendeleev import element

from pymatgen.io.vasp.inputs import Poscar

from pymatgen.core import Structure

from pymatgen.io.cif import CifWriter

from pymatgen.symmetry.analyzer import SpacegroupAnalyzer

from pymatgen.analysis.structure_matcher import StructureMatcher

import os

from collections import Counter

import smact

from smact import element_dictionary, oxidation_states, screening

import re

import math

from functools import reduce

from collections import Counter

elements_ex = [

"He", "Ne", "Ar", "Kr", "Xe", "Rn",

"La", "Ce", "Pr", "Nd", "Pm", "Sm", "Eu", "Gd", "Tb", "Dy",

"Ho", "Er", "Tm", "Yb", "Lu",

"Po", "At", "Fr", "Ra", "Ac", "Th", "Pa", "U", "Np", "Pu",

"Am", "Cm", "Bk", "Cf", "Es", "Fm", "Md", "No", "Lr", "Rf", "Db", 'Sg', 'Bh', 'Hs',

"Mt", 'Ds', 'Rg', 'Cn', 'Nh', 'Fl', 'Mc', 'Lv', 'Ts', 'Og'

]

all_elements = element_dictionary()

def CN(ele,num):

elements = ele

space = smact.element_dictionary(elements)

# We just want the element items from the dictionary

eles = [e[1] for e in space.items()]

# We set a threshold for the stoichiometry of 4

allowed_combinations = smact.screening.smact_filter(eles, threshold=max(num))

possible_comp = []

for i in range(len(allowed_combinations)):

possible_comp.append(list(list(allowed_combinations[i])[2]))

#print(possible_comp)

return(num in possible_comp)

def calculate_overlap_ratio(list1, list2):

counter1 = Counter(list1)

counter2 = Counter(list2)

common_elements = counter1 & counter2

overlap_count = sum(common_elements.values())

total_elements = sum(counter1.values()) + sum(counter2.values())

overlap_ratio = overlap_count / total_elements if total_elements != 0 else 0

return overlap_ratio

def compare_element_period_and_group_differences(element1, element2):

elem1 = element(element1)

elem2 = element(element2)

period1 = elem1.period

group1 = elem1.group.group_id

period2 = elem2.period

group2 = elem2.group.group_id

period_difference = abs(period1 - period2)

group_difference = abs(group1 - group2)

return period_difference + group_difference

def gcd(a, b):

"""计算两个数的最大公约数"""

while b:

a, b = b, a % b

return a

def list_gcd(lst):

"""计算列表中所有数的最大公约数"""

return reduce(gcd, lst)

def normalize_list(lst):

"""将列表中的每个元素除以最大公约数"""

if not lst:

return []

list_gcd_value = list_gcd(lst)

return [x // list_gcd_value for x in lst]

def get_ele_with_same_states(ele):

oxidation_states = element_dictionary([ele])[ele].oxidation_states

elements_with_same_oxidation_states = {symbol: elem for symbol, elem in all_elements.items() if calculate_overlap_ratio(set(elem.oxidation_states),set(oxidation_states)) > 0.3}

return(list(elements_with_same_oxidation_states))

def get_element_types_and_counts(structure):

element_types = list(set(site.specie.symbol for site in structure.sites))

element_counts = {e: sum(1 for site in structure.sites if site.specie.symbol == e) for e in element_types}

return element_types, list(element_counts.values())

def compare_element_period_and_group_differences(element1, element2):

elem1 = element(element1)

elem2 = element(element2)

period1 = elem1.period

group1 = elem1.group.group_id

period2 = elem2.period

group2 = elem2.group.group_id

period_difference = abs(period1 - period2)

group_difference = abs(group1 - group2)

return period_difference + group_difference

def ele_replace(outputpath,filename):

struct = Structure.from_file(filename)

for element in set(struct.species):

element_to_replace = element.symbol

elements_new = get_ele_with_same_states(element_to_replace)

for element_new in elements_new:

if element_new != element_to_replace and element_new not in elements_ex and compare_element_period_and_group_differences(element_new,element_to_replace) < 6:

new_species = []

for site in struct.sites:

if site.specie.symbol == element_to_replace:

new_species.append(element_new)

else:

new_species.append(site.specie.symbol)

new_struct = Structure(struct.lattice, new_species, struct.frac_coords)

ele,num = get_element_types_and_counts(new_struct)

print(ele,num,normalize_list(num))

print('Charge Neutral: ',CN(ele,normalize_list(num)))

if CN(ele,normalize_list(num)):

filename_new = str(outputpath+filename+element_to_replace+element_new)

new_poscar = Poscar(new_struct)

new_poscar.write_file(filename_new)

print('Replacing ',element_to_replace,' with ',element_new,' in ',filename)

current_path = os.getcwd()

os.mkdir('POSCARS')

poscar_files = [f for f in os.listdir(current_path) if f.startswith("POSCAR")]

for filename in poscar_files:

outputpath = 'POSCARS/'

ele_replace(outputpath, filename)

####code3: utils_FailSafe.py

import pandas as pd

import os

import shutil

from pymatgen.io.vasp.inputs import Poscar

from pymatgen.core import Structure

from pymatgen.io.cif import CifWriter

from pymatgen.symmetry.analyzer import SpacegroupAnalyzer

from pymatgen.analysis.structure_matcher import StructureMatcher

import os

from collections import Counter

import smact

import smact.screening

import re

import math

from functools import reduce

import csv

import argparse

def str2list(strele):

ele = strele.strip('[').strip(']').split(',')

for i in range(len(ele)):

ele[i] = ele[i].strip(' ')[1:-1]

return(ele)

def is_subset(A, B):

setA = set(A)

setB = set(B)

return setA <= setB

def gcd(a, b):

while b:

a, b = b, a % b

return a

def list_gcd(lst):

return reduce(gcd, lst)

def normalize_list(lst):

if not lst:

return []

list_gcd_value = list_gcd(lst)

return [x // list_gcd_value for x in lst]

def get_ele_num(cif):

ele,num = [],[]

match = re.search(r'_chemical_formula_sum\s+\s*\'(.*?)\'', cif)

if match:

formula_sum = match.group(1)

else:

print('wrong')

elenum = formula_sum.split(' ')

ele = []

num = []

for n in range(len(elenum)):

ele.append(elenum[n][:-1])

num.append(int(elenum[n][-1]))

return ele, normalize_list(num)

def CN(ele,num):

elements = ele

space = smact.element_dictionary(elements)

# We just want the element items from the dictionary

eles = [e[1] for e in space.items()]

# We set a threshold for the stoichiometry of 4

allowed_combinations = smact.screening.smact_filter(eles, threshold=max(num))

possible_comp = []

for i in range(len(allowed_combinations)):

possible_comp.append(list(list(allowed_combinations[i])[2]))

return(num in possible_comp)

parser = argparse.ArgumentParser(description='Process some CSV files.')

parser.add_argument('--old_data', type=str, required=True, help='Path to the old data file')

parser.add_argument('--new_data', type=str, required=True, help='Path to the new data file')

parser.add_argument('--updated', type=str, required=True, help='Path to the updated data file')

args = parser.parse_args()

old_data = args.old_data

new_data = args.new_data

updated = args.updated

df_old_data = pd.read_csv(old_data)

df_new_data = pd.read_csv(new_data)

print('Previouly generated materials VS new generated materials',len(df_old_data),len(df_new_data ))

id_old = df_old_data['material_id'].values.tolist()

id_ite = df_old_data['Unnamed: 0'].values.tolist()

ele_type_old = df_old_data['elements'].values.tolist()

cifs_old = df_old_data['cif'].values.tolist()

energy_old = df_old_data['formation_energy_per_atom'].values.tolist()

id_new = df_new_data['material_id'].values.tolist()

ele_type_new = df_new_data['elements'].values.tolist()

cifs_new = df_new_data['cif'].values.tolist()

energy_new = df_new_data['formation_energy_per_atom'].values.tolist()

for i in range(len(ele_type_new)):

ele_type_new[i] = str2list(ele_type_new[i])

for i in range(len(ele_type_old)):

ele_type_old[i] = str2list(ele_type_old[i])

removeid_old = []

removeid_new = []

for i in range(len(ele_type_new)):

for j in range(len(ele_type_old)):

if is_subset(ele_type_new[i], ele_type_old[j]):

if energy_new[i]-energy_old[j] < -0.2:

ele_new, num_new = get_ele_num(cifs_new[i])

print(ele_new, num_new)

print('ChargeNeuturalCheck_New: ',CN(ele_new, num_new))

ele_old, num_old = get_ele_num(cifs_old[j])

print(ele_old, num_old)

print('ChargeNeuturalCheck_Old: ',CN(ele_old, num_old))

if CN(ele_new, num_new):

CN1 = 1

else:

CN1 = 0

if CN(ele_old, num_old):

CN2 = 1

else:

CN2 = 0

if CN1 == 1 or CN1+CN2 == 0:

print(i,j)

print(ele_type_new[i], ele_type_old[j])

print(id_new[i], id_old[j]+id_ite[j])

print(energy_new[i],energy_old[j],energy_new[i]-energy_old[j])

print('Eliminating previously generated material:',j)

if j not in removeid_old:

removeid_old.append(j)

if CN1 == 0 and CN2 == 1 and energy_new[i]-energy_old[j] < -0.5:

print(i,j)

print(ele_type_new[i], ele_type_old[j])

print(id_new[i], id_old[j]+id_ite[j])

print(energy_new[i],energy_old[j],energy_new[i]-energy_old[j])

print('***Check CN Eliminating previously generated material:',j)

if j not in removeid_old:

removeid_old.append(j)

if is_subset(ele_type_old[j], ele_type_new[i]):

if energy_new[i]-energy_old[j] > 0.2:

ele_new, num_new = get_ele_num(cifs_new[i])

print(ele_new, num_new)

print('ChargeNeuturalCheck_New: ',CN(ele_new, num_new))

ele_old, num_old = get_ele_num(cifs_old[j])

print(ele_old, num_old)

print('ChargeNeuturalCheck_Old: ',CN(ele_old, num_old))

if CN(ele_new, num_new):

CN1 = 1

else:

CN1 = 0

if CN(ele_old, num_old):

CN2 = 1

else:

CN2 = 0

if CN2 == 1 or CN1+CN2 == 0:

print(i,j)

print(ele_type_new[i], ele_type_old[j])

print(id_new[i], id_old[j]+id_ite[j])

print(energy_new[i],energy_old[j],energy_new[i]-energy_old[j])

print('Eliminating new generated material:',i)

if i not in removeid_new:

removeid_new.append(i)

if CN2 == 0 and CN1 == 1 and energy_new[i]-energy_old[j] > 0.5:

print(i,j)

print(ele_type_new[i], ele_type_old[j])

print(id_new[i], id_old[j]+id_ite[j])

print(energy_new[i],energy_old[j],energy_new[i]-energy_old[j])

print('***Check CN Eliminating new generated material:',i)

if i not in removeid_new:

removeid_new.append(i)

print('Removed: ',removeid_old,removeid_new)

def remove_rows(df, rows_to_remove):

modified_df = df.copy()

modified_df = modified_df.drop(rows_to_remove)

return modified_df

modified_df_old_data = remove_rows(df_old_data , removeid_old)

modified_df_new_data = remove_rows(df_new_data , removeid_new)

combined_data = pd.concat([modified_df_old_data,modified_df_new_data]).reset_index(drop=True)

#deduplicate

removeid_combined = []

cifs = combined_data['cif'].values.tolist()

ids = combined_data['material_id'].values.tolist()

e = combined_data['formation_energy_per_atom'].values.tolist()

structures = []

for i in range(len(cifs)):

structure = Structure.from_str(cifs[i],fmt='cif')

structures.append(structure)

for i in range(len(structures)):

for j in range(i + 1,len(structures)):

matcher = StructureMatcher(ltol = 0.3, stol = 0.2, angle_tol = 10)

if matcher.fit(structures[i], structures[j]):

print(i,j)

print(ids[i],ids[j])

print(e[i],e[j])

if e[i] > e[j]:

removeid_combined.append(i)

else:

removeid_combined.append(j)

combined_data_new = remove_rows(combined_data,removeid_combined)

print('Deduplicate',len(combined_data_new),len(combined_data))

combined_data_new.to_csv(updated,index=False)

#Charge neutral test

data = pd.read_csv(updated)

cifs = data['cif'].values.tolist()

c = 0

for i in range(len(cifs)):

ele,num = get_ele_num(cifs[i])

#print(ele,num)

if CN(ele,num):

c += 1

print(c,len(cifs),round(c/len(cifs),2))

####code5: utils_Gen2Mat.py

import torch

from pymatgen.core import Structure

import numpy as np

import os

import argparse

def generate_cif(lattice, atomnum, atomcord, name, cifpath):

# Create Structure object

structure = Structure(lattice, atomnum, atomcord, to_unit_cell=True)

# Generate .cif files

cif_string = structure.to(fmt="cif")

with open(cifpath+name+".cif", "w") as f:

f.write(cif_string)

def generate_lattice_matrix(lattice_constants, lattice_angles):

# Extract lattice and angles

a, b, c = lattice_constants

alpha, beta, gamma = lattice_angles

# Ang2Rad

alpha_rad = np.radians(alpha)

beta_rad = np.radians(beta)

gamma_rad = np.radians(gamma)

# Get lattice matrix

a1 = a

a2 = b * np.cos(gamma_rad)

a3 = c * np.cos(beta_rad)

b2 = b * np.sin(gamma_rad)

b3 = (c * (np.cos(alpha_rad) - np.cos(beta_rad) * np.cos(gamma_rad))) / np.sin(gamma_rad)

c3 = np.sqrt(c**2 - a3**2 - b3**2)

lattice_matrix = np.array([[a1, 0, 0],

[a2, b2, 0],

[a3, b3, c3]])

return lattice_matrix

def main():

# Set up argument parser

parser = argparse.ArgumentParser(description="Generate CIF files from a checkpoint.")

parser.add_argument('genfile', type=str, help='Path to the .pt checkpoint file')

parser.add_argument('cifpath', type=str, help='Path to the output directory for CIF files')

args = parser.parse_args()

genfile = args.genfile

cifpath = args.cifpath

# Load file

checkpoint = torch.load(genfile, map_location=torch.device('cpu'))

# Extract info

frac_coords = checkpoint['frac_coords'] # (num_evals, N, 3)

atom_types = checkpoint['atom_types'] # (num_evals, N)

lengths = checkpoint['lengths'] # (num_evals, M, 3)

angles = checkpoint['angles'] # (num_evals, M, 3)

num_atoms = checkpoint['num_atoms'] # (num_evals, M)

directory = cifpath

if not os.path.exists(directory):

os.makedirs(directory)

print(f"Directory '{directory}' created.")

else:

print(f"Directory '{directory}' already exists, no action taken.")

for i in range(len(num_atoms.tolist()[0])):

atomnum = num_atoms.tolist()[0][i]

lattice = lengths.tolist()[0][i]

langles = angles.tolist()[0][i]

start = sum(num_atoms.tolist()[0][:i])

atomtype = atom_types.tolist()[0][start:start+atomnum]

atomcord = frac_coords.tolist()[0][start:start+atomnum]

latticem = generate_lattice_matrix(lattice, langles)

generate_cif(latticem, atomtype, atomcord, str(i), cifpath)

if __name__ == "__main__":

main()

####code6: functionsG2DB.py

from pymatgen.core import Structure

from pymatgen.core.lattice import Lattice

from pymatgen.core.periodic_table import Element

from pymatgen.analysis.structure_matcher import StructureMatcher

from pymatgen.symmetry.analyzer import SpacegroupAnalyzer

import numpy as np

import os

import pickle

import pandas as pd

###Funtions

def get_thickness_vac(structure2D):

lattice = structure2D.lattice

c_length = lattice.c

z_coords = [site.frac_coords[2] for site in structure2D.sites]

z_actual_coords = [z * c_length for z in z_coords]

z_min = min(z_actual_coords)

z_max = max(z_actual_coords)

# Handling structures with atoms located at the periodic boundary (vacuum layer in the z-direction)

if (z_max - z_min) > (c_length / 2):

z_actual_coords = []

for z in z_coords:

if z < 0.5:

z_actual_coords.append(z * c_length + c_length)

else:

z_actual_coords.append(z * c_length)

z_min = min(z_actual_coords)

z_max = max(z_actual_coords)

material_thickness = z_max - z_min

vacuum_thickness = c_length - material_thickness

return material_thickness,vacuum_thickness

def scale_vacuum_thickness(structure, target_vacuum_thickness):

lattice = structure.lattice

a, b, c = lattice.abc

material_thickness, current_vacuum_thickness = get_thickness_vac(structure)

new_c_length = material_thickness + target_vacuum_thickness

scale_factor = new_c_length / c

scaled_lattice = lattice.matrix.copy()

scaled_lattice[2] *= scale_factor

frac_coords = structure.frac_coords.copy()

frac_coords[:, 2] /= scale_factor

scaled_structure = Structure(scaled_lattice, structure.species, frac_coords)

return scaled_structure

def unify_crystals(struct):

structS = scale_vacuum_thickness(struct, 15)

structS.scale_lattice(40)

lattice = structS.lattice

coordinates = structS.frac_coords

species = [Element("F") for _ in range(len(coordinates))]

new_struct = Structure(lattice, species, coordinates)

return(new_struct)

def get_difference(structure1,structure2):

step = 0.1

dist = [np.sqrt(0.75)]

for i in range(5):

for j in range(5):

for k in range(5):

#print(i,j,k)

matcher = StructureMatcher(ltol = i * step, stol = j * step, angle_tol = k * step * 90)

if matcher.fit(structure1, structure2):

dist.append(np.sqrt(i**2+j**2+k**2)/10)

return(min(dist))

####code7: eval_HeatOfFormation.py

import argparse

from pymatgen.core import Structure

from matminer.featurizers.base import MultipleFeaturizer

from matminer.featurizers.structure import ChemicalOrdering, MaximumPackingEfficiency, StructureComposition

from matminer.featurizers.composition import Stoichiometry, ElementProperty, ValenceOrbital, IonProperty

import numpy as np

import pandas as pd

import pickle

from os import listdir

from tqdm import tqdm

def build_feature_from_cif(path_cif):

featurizer = MultipleFeaturizer([

ChemicalOrdering(),

MaximumPackingEfficiency(),

StructureComposition(Stoichiometry()),

StructureComposition(ElementProperty.from_preset("magpie")),

StructureComposition(ValenceOrbital(props=['frac'])),

StructureComposition(IonProperty(fast=True))

])

NAME = []

STR = []

ciflist = listdir(path_cif)

for i in tqdm(range(len(ciflist))): # Read POSCAR from file path

try:

cif = ciflist[i]

structuremg = Structure.from_file(path_cif + cif)

STR.append(structuremg)

NAME.append(cif)

except:

print('Error! When featurizing ' + cif)

X = featurizer.featurize_many(STR, ignore_errors=True)

return NAME, X

def main():

# Set up argument parser

parser = argparse.ArgumentParser(description="Featurize CIF files and predict using a ML model.")

parser.add_argument('path_cif_gen', type=str, help='Path to the CIF files directory')

parser.add_argument('npyoutput', type=str, help='Path to the output .npy file')

parser.add_argument('csvoutput', type=str, help='Path to the output .csv file')

parser.add_argument('MLmodel', type=str, help='Path to the trained ML model .pkl file')

args = parser.parse_args()

path_cif_gen = args.path_cif_gen

npyoutput = args.npyoutput

csvoutput = args.csvoutput

MLmodel = args.MLmodel

# Featurize CIF files

name_gen, feature_gen = build_feature_from_cif(path_cif_gen)

np.save(npyoutput, feature_gen)

# Load ML model

with open(MLmodel, 'rb') as file:

gbr = pickle.load(file)

# Predict and save results

pred_gen = gbr.predict(feature_gen)

df = pd.DataFrame()

df['name'] = name_gen

df['Hform'] = pred_gen

df.to_csv(csvoutput, index=False)

if __name__ == "__main__":

main()

####code7: eval_Uniquensee.py

import argparse

import os

import pickle

import pandas as pd

from pymatgen.core import Structure

from pymatgen.core.lattice import Lattice

from pymatgen.core.periodic_table import Element

from pymatgen.analysis.structure_matcher import StructureMatcher

from pymatgen.symmetry.analyzer import SpacegroupAnalyzer

import numpy as np

from functionsG2DB import get_thickness_vac, scale_vacuum_thickness, unify_crystals, get_difference

# Set up argument parser

parser = argparse.ArgumentParser(description='Process some input files and parameters.')

parser.add_argument('--N', type=int, default=10, help='An integer parameter for N.')

parser.add_argument('--folder_path', type=str, default='GenCIFs_/', help='Path to the folder with CIF files.')

parser.add_argument('--outputproto', type=str, default='proto/Gen_prototypesGen_.pkl', help='Path to the output prototype file.')

parser.add_argument('--outputmaterials', type=str, default='proto/Gen_relatedmaterialsGen_.pkl', help='Path to the output materials file.')

parser.add_argument('--csvHoF', type=str, default='data_predicted_HoF_.csv', help='Path to the CSV file with predicted HoF.')

parser.add_argument('--csvoutput', type=str, default='Gen_evaluated_Uniq_.csv', help='Path to the CSV file for evaluated results.')

args = parser.parse_args()

# Access the arguments

N = args.N

folder_path = args.folder_path

outputproto = args.outputproto

outputmaterials = args.outputmaterials

csvHoF = args.csvHoF

csvoutput = args.csvoutput

# The rest of your code using these variables...

# Load structural prototypes in C2DB Ver.2024 and generated prototypes in previously N AL iterations

with open('prototypesC2DB2024.pkl', 'rb') as file:

prototypes_known = pickle.load(file)

prototypes_gen_s = []

for n in range(N):

with open('proto/Gen_prototypesGen'+str(n)+'.pkl', 'rb') as file:

prototypes_gen = pickle.load(file)

with open('proto/Gen_relatedmaterialsGen'+str(n)+'.pkl', 'rb') as file:

relatedmaterials_gen = pickle.load(file)

for i in range(len(relatedmaterials_gen)):

if len(relatedmaterials_gen[i]) > 10:

prototypes_gen_s.append(prototypes_gen[i])

print('Known structures in C2DB:',len(prototypes_known),'Generated structuers in previous generations:',len(prototypes_gen_s))

# Check novelty

Gen_prototypes = []

Gen_relatedmaterials = []

files = os.listdir(folder_path)

print(len(files))

for n in range(len(files)):

file = sorted(files)[n]

state = 0

structure = Structure.from_file(folder_path+'/'+file)

unified_structure = unify_crystals(structure)

matcher = StructureMatcher(ltol = 0.3, stol = 0.2, angle_tol = 10)

for i in range(len(prototypes_known)):

if matcher.fit(prototypes_known[i], unified_structure):

state = 1

print(file,'existed')

break

if state == 0:

for k in range(len(prototypes_gen_s)):

if matcher.fit(prototypes_gen_s[k], unified_structure):

state = 1

print(file,'existed in previous generation')

break

if state == 0:

for j in range(len(Gen_prototypes)):

if matcher.fit(Gen_prototypes[j], unified_structure):

Gen_relatedmaterials[j].append(file)

print(file,'novel material')

state = 1

break

if state == 0:

Gen_prototypes.append(unified_structure)

Gen_relatedmaterials.append([file])

print(file,'novel proto')

with open(outputproto, 'wb') as file:

pickle.dump(Gen_prototypes, file)

with open(outputmaterials, 'wb') as file:

pickle.dump(Gen_relatedmaterials, file)

#Analysis

with open(outputproto, 'rb') as file:

Gen_prototypes = pickle.load(file)

with open(outputmaterials, 'rb') as file:

Gen_relatedmaterials = pickle.load(file)

N_new = 0

for i in range(len(Gen_relatedmaterials)):

#print(len(Gen_relatedmaterials[i]))

N_new += len(Gen_relatedmaterials[i])

print(N_new)

sorted_list = sorted(Gen_relatedmaterials, key=len, reverse=True)

count = 0

percent = []

materials = []

for sublist in sorted_list:

if len(sublist) > 5:

count += len(sublist)

#print(count,round(count/1057,2))

#percent.append(sublist[0])

print(len(sublist),round(len(sublist)/1057,3),sublist[0],sublist[4])

s_gen = []

for i in range(len(Gen_relatedmaterials)):

if len(Gen_relatedmaterials[i]) > 5:

s_gen.append(Gen_prototypes[i])

percent.append(Gen_relatedmaterials[i][0])

materials.append(Gen_relatedmaterials[i])

print(len(percent))

print(len(materials))

print(len(s_gen))

#Calculate uniquness

dist_gen = []

for j in range(len(percent)):

print(j,percent[j])

struture = s_gen[j]

#print(len(struture.frac_coords))

dists = []

for i in range(len(prototypes_known)):

#print(i)

if len(struture.frac_coords) == len(prototypes_known[i].frac_coords):

#print(struture,screened_prototypes[i])

d = get_difference(struture,prototypes_known[i])

dists.append(d)

print(d)

for i in range(len(prototypes_gen_s)):

#print(i)

if len(struture.frac_coords) == len(prototypes_gen_s[i].frac_coords):

#print(struture,screened_prototypes[i])

d = get_difference(struture,prototypes_gen_s[i])

dists.append(d)

print(d)

print('Generated proto VS existed protos:',min(dists))

dist_gen.append(min(dists))

pdtemp = pd.read_csv(csvHoF)

uniq = []

ELEM = []

ids = pdtemp['name'].values.tolist()

for i in range(len(ids)):

state = 0

for j in range(len(dist_gen)):

if ids[i] in materials[j]:

uniq.append(dist_gen[j])

#print(dist_gen[j])

state = 1

if state == 0:

uniq.append(0)

structure = Structure.from_file(folder_path+ids[i])

ele = structure.species

for j in range(len(ele)):

ele[j] = str(ele[j])

ELEM.append(ele)

pdtemp['uniqueness'] = uniq

pdtemp['elements'] = ELEM

pdtemp.to_csv(csvoutput, index=False)

####BASH Codes sym_trans.py

conda init

conda activate phonon

for file in POSCAR*; do

if [[ -f "$file" ]]; then

phonopy --symmetry --tolerance=1.0e-1 -c "$file"

if [[ -f "PPOSCAR" ]]; then

mv PPOSCAR "$file"

echo "Renamed PPOSCAR to $file"

else

echo "PPOSCAR file not found for $file"

fi

else

echo "$file is not a regular file"

fi

done

rm BPOSCAR phonopy_symcells.yaml

####COMMANDS to RUN .py

#from eval_gen.pt to cifs

python utils_Gen2Mat.py eval_genAL1.pt GenCIFsAL1/

#evaluate the heat of formation with a trained ML model

python eval_HeatOfFormation.py GenCIFsAL1/ MAGPIEAL1.npy data_predicted_HoF_AL13.csv trained_HoF_model.pkl

#evaluate the uniqueness of generated materials

python script.py --N 0 --folder_path 'GenCIFsAL1/' --outputproto 'proto/Gen_prototypesGen1.pkl' --outputmaterials 'proto/Gen_relatedmaterialsGen1.pkl' --csvHoF 'data_predicted_HoF_AL1.csv' --csvoutput 'Gen_evaluated_UniqAL1.csv'

#evaluate the energy above hull of generated materials compared to samples in C2DB

python eval_ehull.py 'Gen_evaluated_UniqAL1.csv' 'Gen_predictH_EhullAL1.csv'

#sampling materials with highst novelty stability and diversity

python utils_ALsampling.py --source_fold 'GenCIFsAL1/' --target_fold 'GensampledAL1/' --gendata 'Gen_predictH_EhullAL1.csv' --gen_magpie 'MAGPIEAL1.npy'

#check if generated materials are new

python utils_CheckNovelty.py --path_selected 'selectAL1ER/' --path_target 'screenAL1ER/' --task ALER --ite 1

#transform screened materials into CDVAE input .csv format

python utils_CIF2CSV.py --path_screened_materials 'screenAL1/' --file_data '../AL1/Fine_cal/energy_per_atom.csv' --file_dataref '../AL1/Fine_cal/Ehull_Cont/energy_per_atom.csv' --csv_file 'outputR1.csv' --task 'AL' --ITE '1'

#fail safe, remove dupilicates and materials

python utils_FailSafe.py --old_data 'output_updated1ER.csv' --new_data 'outputR1.csv' --updated 'output_updated1.csv'
