# Supplementary material for: Continuous discovery of novel 2D materials via dual active learning-driven generative models
Source: Natl Sci Rev. 2026 Feb 12;13(7):nwag101. doi: 10.1093/nsr/nwag101 (PMC13107964; doi:10.1093/nsr/nwag101)
Supplement: nwag101_Supplemental_Files [file nwag101_supplemental_files.zip › Teaser text.docx]

This study introduces DuALGen, which couples generative and predictive active learning loops to explore unknown chemical spaces, discovering over 10,000 novel, stable 2D materials with high-performance candidates for electronic applications.
